# Enhanced probiotic potential of *Lactobacillus kefiranofaciens* OSU-BDGOA1 through co-culture with *Kluyveromyces marxianus* bdgo-ym6

**DOI:** 10.3389/fmicb.2023.1236634

**Published:** 2023-08-03

**Authors:** Brianda D. González-Orozco, Erica Kosmerl, Rafael Jiménez-Flores, Valente B. Alvarez

**Affiliations:** Department of Food Science and Technology, The Ohio State University, Columbus, OH, United States

**Keywords:** co-culture, probiotics, exopolysaccharides (EPS), kefir, *in vitro* digestion, heat-killed *Salmonella* Typhimurium cells (HKSC)

## Abstract

**Introduction:**

Due to the increasing consumer demand for the development and improvement of functional foods containing probiotics, new probiotic candidates need to be explored as well as novel means to enhance their beneficial effects. *Lactobacillus kefiranofaciens* OSU-BDGOA1 is a strain isolated from kefir grains that has demonstrated probiotic traits. This species is the main inhabitant of kefir grains and is responsible for the production of an exopolysaccharide (EPS) whit vast technological applications and potential bioactivities. Research has shown that interkingdom interactions of yeast and lactic acid bacteria can enhance metabolic activities and promote resistance to environmental stressors.

**Methods:**

Comparative genomic analyses were performed to distinguish OSU-BDGOA1 from other strains of the same species, and the genome was mined to provide molecular evidence for relevant probiotic properties. We further assessed the cumulative effect on the probiotic properties of OSU-BDGOA1 and *Kluyveromyces marxianus bdgo-ym6* yeast co-culture compared to monocultures.

**Results:**

Survival during simulated digestion assessed by the INFOGEST digestion model showed higher survival of OSU-BDGOA1 and *bdgo-ym6* in co-culture. The adhesion to intestinal cells assessed with the Caco-2 intestinal cell model revealed enhanced adhesion of OSU-BDGOA1 in co-culture. The observed increase in survival during digestion could be associated with the increased production of EPS during the late exponential and early stationary phases of co-culture that, by enhancing co-aggregation between the yeast and the bacterium, protects the microorganisms from severe gastrointestinal conditions as observed by SEM images. Immune modulation and barrier function for recovery and prevention of flagellin-mediated inflammation by *Salmonella* Typhimurium heat-killed cells (HKSC) in Caco-2 cells were also measured. OSU-BDGOA1 in mono- and co-culture regulated inflammation through downregulation of pro-inflammatory cytokine expression and increased membrane barrier integrity assessed by TEER, FD4 permeability, and expression of tight junctions.

**Discussion:**

The results of the study warrant further research into the application of co-cultures of yeast and LAB in functional probiotic products and the potential to increase EPS production by co-culture strategies.

## 1. Introduction

Probiotic microorganisms are widely consumed around the world and have gained popularity for their potential to improve human health (Ailioaie and Litscher, 2021; Cunningham et al., 2021). The most recent definition of probiotics is “live microorganisms, which when administered in adequate amounts confer a health benefit on the host” (Hill et al., 2014). Probiotics exert their beneficial effects through different mechanisms including supporting the gut microbiota, producing antibacterial peptides, outcompeting pathogens for nutrients and preventing their adhesion to intestinal cells, modulating the immune system, enhancing intestinal barrier function, and reducing inflammation. However, these properties are strain-specific and a comprehensive genetic and phenotypic characterization of potential probiotic strains and their associated health benefits is required (Liu et al., 2018; Raheem et al., 2021).

The most widely used probiotic bacteria for human consumption include members of the *Lactobacillus* and *Bifidobacterium* genera. Further, dairy products are the most common vehicles for delivery to consumers due to their complex nutritional profiles (Gullo and Zotta, 2022). With increasing consumer demand for the development and improvement of functional foods containing probiotics, new probiotic candidates need to be explored as well as novel means to enhance their probiotic properties.

Kefir is a fermented dairy product originating from the Caucasus mountains that has been consumed for centuries due to its associated health benefits. Traditional kefir is produced from the fermentation of milk with kefir grains, which are complex microbial communities of lactic acid bacteria (LAB), acetic acid bacteria, and yeast embedded in an exopolysaccharide (EPS) matrix known as kefiran (Nejati et al., 2020). During kefir production, EPS-producing bacteria play an important role in traditional kefir production, as they acidify and improve the viscosity and texture of the fermented product. Recently, EPS from probiotic bacteria are receiving renewed interest due to their potential functional and bioactive properties. Several studies have demonstrated the biological activities of EPS, including prebiotic effects, modulation of the host immune response, antagonism of pathogens in the gut, and increasing barrier integrity (Jurášková et al., 2022; Sørensen et al., 2022). However, due to the complexity of the kefir grains and unique fermentation dynamics, the production of a stable kefir product remains unfeasible at a commercial level (Yilmaz et al., 2022). Therefore, there is a significant opportunity to isolate, identify, and characterize specific EPS-producing kefir strains with functional benefits, and a need for understanding the detailed mechanisms underlying their probiotic effects.

*Lactobacillus kefiranofaciens* is the main habitant of kefir grains and is responsible for the production of a unique EPS known as kefiran. Kefiran has multiple potential technological applications as a thickener and texture-modifier, and also exhibits various bioactivities, including antibacterial, antioxidant, and immune regulatory properties (Vinderola et al., 2006; Wang X. et al., 2018). Several strains of *L. kefiranofaciens* isolated from various kefir grain origins have shown probiotic traits; however, genotypic characterization is restricted due to the limited number of genome sequences available. *Lactobacillus kefiranofaciens* OSU-BDGOA1 (GenBank number: JARJVW01) is a strain previously isolated by our group and has shown activity against indicator microorganisms, resistance to gastrointestinal tract conditions, lack of blood hemolysis, and absence of antibiotic resistance to common antibiotics (González-Orozco et al., 2023).

*Kluyveromyces marxianus* is the predominant yeast found in kefir grains and plays a crucial role in the production of desirable flavor compounds (e.g., ethyl acetate and phenylethanol) in milk kefir. Relative to other yeast species, such as *Saccharomyces cerevisiae*, *K. marxianus* has several physiological advantages due to its ability to ferment a wide variety of sugars and its thermotolerance, with a maximum growth temperature of 52°C (Ahtesh et al., 2018). Furthermore, *K. marxianus* strains have exhibited probiotic traits (Maccaferri et al., 2012; Diosma et al., 2014). *Kluyveromyces marxianus* bdgo-ym6 (GenBank number: MZ927822.1) was previously isolated by our group from kefir grains. In a preliminary screening, this strain showed potential in resisting gastrointestinal tract conditions, surface hydrophobicity, auto-aggregation ability, and proteolytic activity (González-Orozco et al., 2023).

In natural environments, including the human gut, microorganisms exist as part of complex communities consisting of hundreds or thousands of strains that form unique networks of relationships. For instance, a consortium of microorganisms may provide crucial benefits like the division of labor toward a metabolic purpose where more than one strain is involved (Canon et al., 2020b). The value of co-cultivation has been exploited in many fields including the production of vitamins and enzymes, wastewater treatments, in the bioenergy sector, and to enhance flavor and rheological properties of fermented foods (Kapoore et al., 2022).

In food applications, usually microbial consortia of different probiotic LAB are used with the purpose of enhancing probiotic traits and metabolic capacities. Bolla et al. (2013) showed that the co-culture of lactic acid bacteria (*L. plantarum, L. kefir, L. lactis*) and yeast (*K. marxianus, S. cerevisiae*) reduced the levels of a *C. difficile-*induced enterocolitis in a hamster model. Another co-culture of kefir microorganisms (*Lactiplantibacillus plantarum* CIDCA 8327, *Lentilactobacillus kefiri* CIDCA 8348, and *K. marxianus* CIDCA 8154) showed positive effects against *Salmonella* spp. in Caco-2/TC7 cells in an *in vitro* model (Londero et al., 2015). Overall, these studies suggest that the use of a co-culture of probiotic strains could trigger beneficial effects through metabolic cooperation through division of labor and removal of inhibitory metabolites (Canon et al., 2020a). However, designing and controlling a synthetic consortium of different LAB to achieve a metabolic goal is challenging due to competitive exclusion, in which two non-interbreeding populations competing for the same ecological niche and nutritional requirements cannot coexist (Grandel et al., 2021). As many LAB utilize the same nutrients, combining strains with similar metabolic dependencies in a co-culture can result in the faster growing population completely overtaking the slower growing population. Therefore, understanding the positive microbial interactions among members of the consortium is essential and a consortia of a single LAB strain and a single yeast with different ecological niches may overcome these challenges (Giri et al., 2020).

Interkingdom consortia of yeasts and LAB are of key importance in natural fermented products such as kefir, kimchi, beer, kombucha, sourdough, etc. (Tamang et al., 2016). As shown by Ponomarova et al. (2017), by-products of yeast metabolism such as amino acids and small peptides can render new metabolic niches for LAB. Additionally, lactic acid accumulation during fermentation inhibits growth of LAB and results in a decrease in productivity of metabolites like EPS. Sieuwerts et al. (2008) showed that in kefir grains *S. cerevisiae* raises the pH by utilizing the lactic acid produced by *L. kefiranofaciens* as a carbon source and enables prolonged growth of the bacterium. Bertsch et al. (2019) reported that co-culturing *S. cerevisiae* with *Lactobacillus delbrueckii* increased kefiran production and bacteriocin (nisin) production.

Therefore, we hypothesized that a co-culture of *L. kefiranofaciens* OSU-BDGOA1 and *K. marxianus* bdgo-ym6 will enhance probiotic traits through positive symbiotic microbial interactions using a model system. In the present study, we assessed the cumulative effect of co-culturing kefir microorganisms *L. kefiranofaciens* OSU-BDGOA1 and *K. marxianus* bdgo-ym6 compared to single strains on the resistance to simulated digestion, adhesion to epithelial cells, EPS production, antibacterial activity, and regulation of the gut-associated immune system and membrane barrier upon flagellin-mediated inflammation by *Salmonella enterica* serovar Typhimurium LT2 heat-killed cells in a Caco-2 intestinal cell model.

## 2. Materials and methods

### 2.1. Genomic comparison of *Lactobacillus kefiranofaciens* strains

For a comparative analysis between *L. kefiranofaciens* strains, the genome sequence of OSU-BDGOA1 was compared to three other *L. kefiranofaciens* strains (1,207, ZW3, LKK75) from GenBank (Supplementary Table S1). To estimate differential gene content of the genomes and other comparison metrics, we used the comparative genomics platform EDGAR (Blom et al., 2009).

### 2.2. Genotypic characterization based on screening of targeted functions

*Lactobacillus kefiranofaciens* OSU-BDGOA1 was previously identified by PCR amplification of a major part of the 16 s rRNA gene, as described in González-Orozco et al. (2023). Identity confirmation was performed by whole genome sequencing (BioSample ID: SAMN31370630). The genome of OSU-BDGOA1 was mined for genes associated with bacteriocin production using BAGEL4 (van Heel et al., 2018) and for potential phages using Prophage Hunter (Song et al., 2019). Genomic islands of virulence factors, resistance genes, and pathogen-associated genes were tracked with IslandViewer4 (Bertelli et al., 2017) and compared with other *L. kefiranofaciens* strains (1,207, ZW3, and LKK75). Additionally, genes encoding for specific functions (i.e., the genes encoding carbohydrate metabolism, genes for resistance to gastric tract conditions, and EPS production) were also mined from the annotation with IslandViewer4 (Supplementary Table S2).

### 2.3. Co-culture growth conditions

Preliminary experiments for *L. kefiranofaciens* and *K. marxianus* growth in co-culture showed fitness of growth of both microorganisms in co-culture at 30°C in aerobic conditions when cultivated in MRS broth (Sigma Aldrich, St. Louis, MO) (Supplementary Table S3). *Lactobacillus kefiranofaciens* and *Kluyveromyces marxianus* strains were stored with 20% glycerol (Thermo Fisher, Waltham, MA) in De Man Rogosa Sharpe (MRS) broth and Yeast Extract Peptone Dextrose (YEPD) broth, respectively, at −80°*C. prior* to each experiment, the strains were activated from the glycerol stocks in 5 mL of either MRS or YEPD broth and incubated aerobically for 16 h at 30°C. Liquid cultures were sub-cultured twice before the start of all experiments.

### 2.4. Survival during *in vitro* INFOGEST digestion

Survival during digestion was assessed following the static INFOGEST 2.0 digestion model consisting of oral, gastric, and intestinal phases (Brodkorb et al., 2019) with slight modifications. All digestion reagents were purchased from Sigma Aldrich. *L. kefiranofaciens* and *Kluyveromyces marxianus* were pre-cultured as described previously in MRS and YEPD broth, respectively. Stationary phase cultures were centrifuged (4,200 × g, 4°C, 10 min), washed twice with saline solution (0.85% NaCl, pH 7.0) and resuspended to an optical density (OD) of 0.1 at 600 nm, corresponding to approximately 10^8^ CFU/mL for bacteria and 10^6^ CFU/mL for yeast. The OD = 0.1 suspensions were inoculated at a ratio of 10% of the final volume in MRS broth for each monoculture control. For the co-culture, the OD-adjusted suspensions were mixed 1:1 (v/v) and inoculated at a ratio of 10% of the final volume in MRS broth based on the ratio of these strains present in kefir. The cultures were then incubated aerobically for 16 h at 30°C. Immediately before the start of simulated digestion, the OD of each monoculture and the co-culture were adjusted to 0.1 at 600 nm in saline solution and 5 mL of each suspension was used at the start of digestion. At the end of the gastric and intestinal phases, the digesta was centrifuged (3,500 × g, 4°C, 10 min) and the supernatant was removed, the microbial pellet was resuspended in 5 mL of saline solution and serial dilutions were made. CFUs of the monoculture and co-culture at the end of the gastric and intestinal phases were counted by plating onto selective media: MRS supplemented with 0.02 g/L cycloheximide were used to selectively estimate *L. kefiranofaciens* and potato dextrose agar (PDA) supplemented with 0.1 g/L chloramphenicol was used to selectively estimate *K. marxianus* yeast in the co-culture. For monocultures MRS agar was used for *L kefiranofaciens* and PDA for *K. marxianus*. Plates were incubated for 2 days at 30°C before counting. Survival percentage was determined as follows:


Survival%=CFUmLof survivors in each phaseCFUmLpre−digestion control×100

### 2.5. Caco-2 cell culture

The human colorectal adenocarcinoma Caco-2 cell line (HTB37, American Type Culture Collection) was used as a model of intestinal epithelium cells. Cells were seeded in T-75 cm^2^ flasks (Corning) with complete Dulbecco’s modified Eagle’s medium (cDMEM) supplemented with 10% heat-inactivated fetal bovine serum, 1% penicillin–streptomycin (100 units/mL penicillin and 100 units/mL streptomycin), 1% non-essential amino acids (100X), and 1% 200 mM L-glutamine in a humidified atmosphere of air and CO_2_ (95:5, vol/vol) for 4 h at 37°C. Cells were passaged by trypsinization at 70–90% confluence (6 ± 1-day post-seeding) and passages between 23 and 35 were utilized for the experiments. Spent medium was replaced every 48 h. Media was replaced with serum- and antibiotic-free media one day prior to all experiments. All cell culture reagents were purchased from Thermo Fisher (Gibco).

### 2.6. Adhesion to Caco-2 cells

Caco-2 cells were seeded at a density of 1 × 10^5^ cells per well in a 12-well culture plate (Corning) and cultured for 14 days post-confluency for adequate differentiation. Caco-2 cells were incubated with 1.5 mL of mono- and co-culture stationary-phase suspensions (OD_600_ = 0.1) for 4 h at 37°C. To account for changes in microbial growth in the presence of cDMEM, both mono- and co-culture suspensions without Caco-2 cells were simultaneously incubated in cDMEM. These suspensions served as the control for added microorganisms. At the end of the incubation time, Caco-2 cells were washed two times with sterile PBS to remove non-adherent bacteria and lysed with 1% Triton X-100 (v/v) for 30 min at 4°C to release adherent microorganisms. Samples were centrifuged (8,000 × g, 4°C, 10 min) and washed twice before resuspending in 1.0 mL of saline solution. Adhesion of the monoculture and co-culture were determined by plating serial dilutions on selective media as explained above. Adhesion percentage was determined as follows:


Adhesion%=CFUmLof adhered microorganismsCFUmLadded microorganisms×100.

Adhesion assays were carried out in three independent experiments, each containing three replicates.

### 2.7. Growth and exopolysaccharides (EPS) production

For the production of EPS in co-culture and monoculture of *L. kefiranofaciens*, we determined the EPS concentration at different time points (4, 8, 12, 20, 24, and 48 h) during incubation at 30°C. At the same time, the growth curves were prepared by incubating 20 μL of mono- and co-culture suspensions (OD_600_ = 0.1) in 180 μL MRS media in a sterile 96-well plate in triplicate. The OD_600_ was recorded every 2 h over the aerobic incubation time with 5 s of low-speed shaking prior to each read.

Based on previous reports that address the overestimation of bacterial EPS production by the subsequent extraction of medium constituents such as beef and yeast extract during EPS characterization and quantification, we adjusted the methodology to account for these limitations. Specifically, we use a modified version of Casein Glucose Broth (mCGB) replacing yeast extract with yeast nitrogen base (YNB) and peptone with casein acid hydrolysate (Sigma Aldrich) according to Alhudhud et al. (2014). We also determined that the mCGB was able to support the growth of the co-culture and monocultures. The EPS extraction was performed using the methods of Ferrari et al. (2022) with modifications. Briefly, 9 mL of fresh medium was inoculated with 1 mL of co-culture and monoculture suspensions (OD_600_ = 0.1) and incubated aerobically at 30°C. Separate tubes were prepared for each time point. At each time point, cells were harvested by centrifugation at (4,200 × g, 4°C, 10 min), the supernatants were treated with trichloroacetic acid (TCA) at a final concentration of 12% (v/v) and incubated at −20°C for 30 min. After centrifugation (7,000 × g, 4°C, 20 min), the pH of the supernatants was adjusted to ~7 by addition of 5 M NaOH, the polysaccharides were precipitated with two volumes of EtOH after overnight incubation at −20°C. Pellets were collected by centrifugation (9,000 × g, 4°C, 30 min) and resuspended in MilliQ water (18.2 MΩ • cm).

Total EPS in mg/mL was determined by subtracting the total sugar content assessed by the phenol sulfuric acid assay with glucose as a standard (Dubois et al., 1956) from the reducing sugars estimated by the DNSA (Dinitrosalicylic acid) method (Gonçalves et al., 2010). Additionally, protein quantification was performed with the Pierce micro-BCA kit (Thermo Fisher).

### 2.8. Monosaccharide composition and quantification by HPLC-CAD

EPS were extracted as previously described from 200 mL of the co-culture and monoculture in mCGB media after 16 h of incubation at 30°C. Following extraction, EPS were dialyzed with a 3.5 kDa MWCO Amicon Ultra-15 Centrifugal Filter Unit (Millipore) and lyophilized. EPS were hydrolyzed to monosaccharides using the method of Zajšek et al. (2013) with modifications. Briefly, 25 mg of lyophilized EPS powder were dissolved in 2 mL of MilliQ water and sonicated for 10 min to completely dissolve the sugars. Next, 1-mL of the sample was transferred to a 2 mL tube and treated with 0.5 mL of 0.5 M H_2_SO_4_ for 1.5 h at 100°C. Samples were diluted by adding 1.5 mL of water, pH was adjusted with 5 M NaOH, and further diluted to a final concentration of 4 mg/mL in MilliQ water.

High-purity HPLC-grade solvents, triethylamine, ammonium acetate, and the carbohydrates (glucose, galactose, and mannose) analytical standards were obtained from either Sigma-Aldrich or Fisher Scientific. The analysis was conducted on an XBridge Amide 3.5 μm (4.6 × 250 mm) column (Waters) equipped with a guard column of the same filling on a Dionex Ultimate 3,000 HPLC system with a charged aerosol detector (CAD; Thermo Fisher). A column temperature of 80°C was maintained throughout the run. The volume injected was 50 μL of samples prepared in 60% (v/v) ACN. Elution of monosaccharides was performed at a flow rate of 0.5 mL/min with a multi-step-gradient using two mobile phases. Solvent B consisted of 90% ACN +10% H_2_O + 0.2% TEA +25 mM ammonium acetate. Solvent A consisted of 50% ACN + 50% H_2_O with 0.2% v/v TEA and 25 mM of ammonium acetate. The isocratic conditions used were as follows: 90% Solvent B (t = 0–32 min), followed by column washing and equilibration. Separation and elution of the carbohydrates analyzed occurred within the first 20 min. The CAD evaporator temperature was set to 35°C with a data collection rate of 10 Hz and filter constant of 1.0. HPLC-grade glucose (D-Glu), galactose (D-Gal), and mannose (D-Man) standards were used for quantification. The system was controlled by Chromeleon 7.2.9 software. The final sugar quantification was normalized by CFU/mL of the bacterium.

### 2.9. Scanning electron microscopy

To visualize the EPS produced during the mono and co-culture and analyze their physical and structural properties, the stationary-phase cultures of the mono and co-culture and the lyophilized EPS were imaged using a Schottky Field Emission Scanning Electron Microscope (Hitachi High Technologies America, Inc., Schaumburg, IL, United States) at the Molecular and Cellular Imaging Center (MICI) at The Ohio State University (Wooster, OH). For the fixation process, the microbial pellets were resuspended in 1 mL of fixative (3% glutaraldehyde, 2% paraformaldehyde in 0.1 M of PBS buffer) and incubated at 25°C for 2 h. After centrifugation, the obtained pellet was resuspended in PBS buffer for further addition to a silicon chip and dehydration in acetone.

### 2.10. Antibacterial activity and sensitivity of antibacterial substances to catalase and proteases

The agar well diffusion method was used to test the antibacterial activity of cell free supernatants (CFS) of the co-culture and monocultures against indicator strains (*Escherichia coli* ATCC 25922 and *Listeria innocua* ATCC 51742). Briefly, CFS were collected by centrifugation (4000 × g, 10 min, 4°C), adjusted to a pH of 7.00 ± 0.05 using 5 M NaOH, and filtered through a nitrocellulose membrane (0.22 μm). Tryptic soy agar plates were overlaid with 10 mL of soft TSA medium (0.75%) and seeded with 10 μL of indicator strains (~10^6^–10^7^ CFU ml^−1^). After solidification, 9-mm diameter wells were cut from the agar and 50 μL of the CFS were spotted and maintained at 25°C for 2 h to allow diffusion before incubation at 37°C for 16–18 h. The agar plates were then examined to detect the formation of a clear zone of inhibition around the wells. To determine that the antibacterial activity was not associated with production of hydrogen peroxide and the susceptibility of antibacterial substances to proteases, supernatants were treated with catalase, and trypsin according to the methods followed by Wang et al. (2019). CFS from each strain were incubated for 2 h at 37°C with catalase (3000 U/mg) and trypsin (250 U/mg). The enzymes were used at a final concentration of 1 mg/mL in 50 mM sodium phosphate buffer (pH = 7.00). After incubation, the enzymes were inactivated at 80°C for 10 min. After treatment, the antibacterial ability was determined as described before. A heat control of the CFS was also evaluated to determine sensitivity of the CFS to the heat treatment.

### 2.11. Study of the regulation of the immune response and membrane barrier in Caco-2 cells

#### 2.11.1. Inflammation induction by heat-killed *Salmonella* Typhimurium cells (HKSC)

*Salmonella enterica* serovar Typhimurium LT2 heat-killed cells (HKSC, kindly donated by Dr. Ahmed Yousef, The Ohio State University, Columbus, OH, United States) were used to study the effect on the immune responses of the mono- and co-cultures at prevention and recovery upon flagellin-mediated inflammation by *Salmonella* Typhimurium in Caco-2 cells. *Salmonella* Typhimurium cells were activated from glycerol stocks in TSA (1.5%) plates. Liquid cultures were sub-cultured twice before the inactivation experiment. Pellets OD_600nm_ = 0.5 were recover by centrifugation, washed twice, and resuspend in PBS buffer. HKSC were prepared by heat treatment at 65°C for 1 h. To ensure complete cell inactivation the resulting suspension was plated in TSA plates and incubated for 24 h at 37°C in aerobic conditions. The inflammatory response was measured by the gene expression of pro- and anti-inflammatory cytokines (*Il-8, Mcp-1, Il-6*, and *Il-10*), as these are key modulators of the inflammatory response. Since the time of administration of probiotics is crucial in determining their response (Liu et al., 2018; Milner et al., 2021), the effect of the mono and co-culture before (preventive group) and after (recovery group) the HKSC-induced inflammation was evaluated.

To determine the concentration of HKSC that will induce an inflammation response in Caco-2 cells, preliminary experiments testing different concentrations and times of exposure were conducted and the inflammation response evaluated by expression of pro-inflammatory cytokines. Cytotoxicity to the mammalian cells for all the treatments was also determined with the lactose dehydrogenase (LDH) assay using a commercial kit (Sigma-Aldrich) (data not shown).

**Figure 1:**
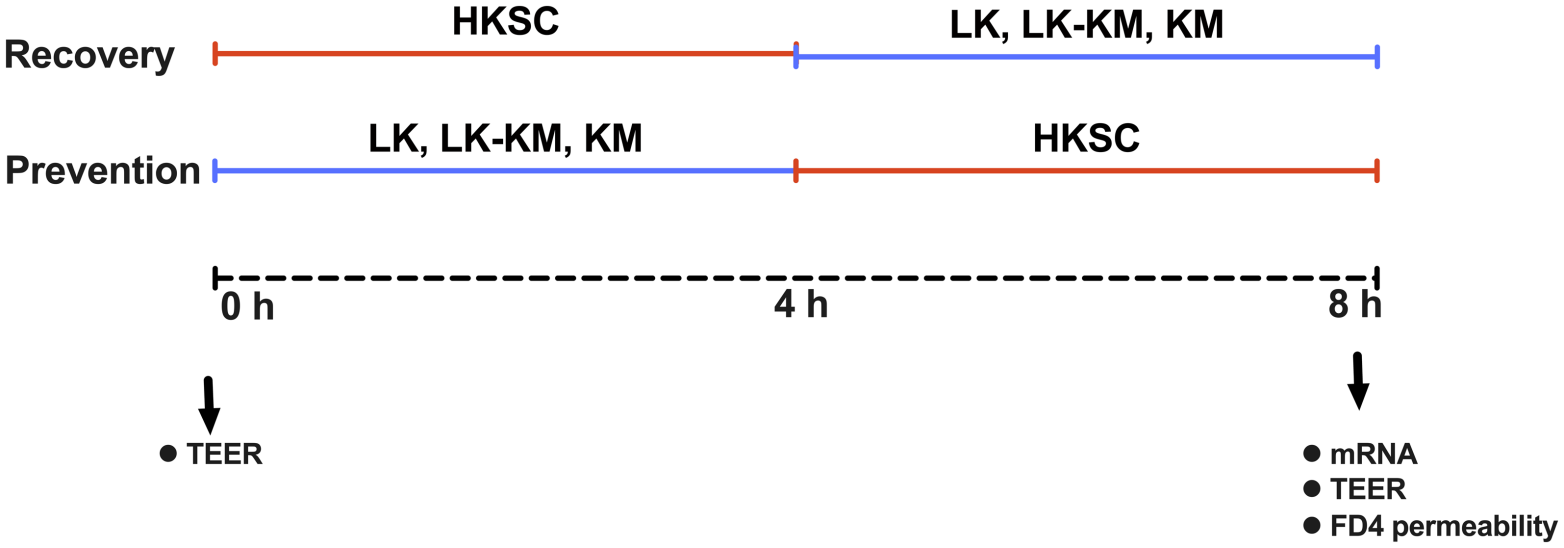
Experimental design used to test the immune regulation capacity of *Lactobacillus kefiranofaciens* (LK) and *Kluyveromyces marxianus* (KM) in mono- and co-culture at prevention and recovery upon flagellin-mediated inflammation by heat-killed *Salmonella* Typhimurium cells (HKSC) in Caco-2 intestinal cells model. Recovery group: 4 h of inflammation induction with HKSC followed by 4 h treatment with monocultures (LK, KM) and co-culture (LK-KM). Prevention group: 4 h treatment with monocultures (LK, KM) and co-culture (LK-KM) followed by 4 h of inflammation induction with HKSC.

For the recovery group, fully-differentiated Caco-2 cells seeded in 24 well plates (Corning) were washed with sterile DPBS and replaced with 500 μL of a suspension HKSC (MOI ≈ l00) in serum- and antibiotic-free media and incubated for 4 h at 37°C. Subsequently, 500 μL of stationary phase suspensions of mono- and co-cultures in serum- and antibiotic-free media were added to the wells and incubated for an additional 4 h. For the preventive group, the stationary phase suspensions of mono- and co-culture were first added to the cells, incubated for 4 h at 37°C, and then treated with 500 μL of HKSC (MOI ≈ l00) in serum- and antibiotic-free media for an additional 4 h. At the end of the experiment, Caco-2 cells were washed twice with DPBS (pH 7.0) and harvested with 200 μL of Trizol reagent (Sigma-Aldrich). Samples were stored at −80°C until further RNA extraction. Wells treated with HKSC for 8 h were used as positive controls, untreated Caco-2 cells in serum- and antibiotic-free media were used as negative controls. Additionally, to account for the effect of the co-culture and respective monocultures and in the mammalian cells, controls without HKSC were also evaluated. The layout of the experimental design, including the different treatments and time points is shown in Figure 1.

#### 2.11.2. Gene expression by qPCR

RNA was extracted from Caco-2 cells after treatment using Trizol according to the manufacturer’s protocol (Sigma Aldrich). Reverse transcription of RNA was performed with the iScript Reverse Transcription Supermix kit (Bio-Rad) and the cDNA was subsequently diluted ten times. Amplification of primer-specific cDNA was achieved using the SsoAdvanced Universal SYBR Green Supermix (Bio-Rad). Primers for the human inflammatory cytokines (*Il-8, Mcp-1, Il-6*) and anti-inflammatory cytokines (*IL-10*) were amplified using PrimerPCR SYBR Green Assays (Bio-Rad) in a CFX96 Touch System with expression being normalized to actin. Primer specificity and efficiency was assessed from the melt curves. Analysis was performed using CFX Maestro Software (Bio-Rad).

#### 2.11.3. Determination of monolayer integrity of Caco-2 cells

Experiments to determine the effect of mono- and co-culture on the prevention and recovery of HKSC-induced disruptions of monolayer integrity were performed on 6-well membrane inserts with a 0.4 μm PET tracked-etched membrane (Corning). Inserts were seeded apically with 1.5 mL of cDMEM containing 2 × 10^5^ cells per insert and maintained for 21 days for complete differentiation of Caco-2 cells. Only the monolayers with epithelial resistance reaching ≥300 Ω cm^2^ were used for experiments (21 days post-confluency).

#### 2.11.4. Trans-Epithelial Electrical Resistance (TEER) and paracellular permeability

Caco-2 cells were washed with sterile DPBS and 750 μL of HKSC (MOI ≈ l00) or co-culture and monocultures suspensions in serum- and antibiotic-free media were added apically according to the experimental design and incubated for 4 h at 37°C. After the first incubation time, additional 750 μL suspensions of HKSC or monocultures and co-culture were added to the inserts and incubated for an additional 4 h at 37°C.

TEER was measured using a EVOM Epithelial Voltohmmeter with an STX2 probe (Millipore) in sterile DPBS after a washing step to remove old medium breakdown products that can affect the TEER measurements as described by Hubatsch et al. (2007). TEER change was expressed as the ratio of the TEER at the end of the treatments compared to the initial value. The paracellular permeability of the Caco-2 monolayers was determined by the transport of fluorescein isothiocyanate (FITC)-labeled dextran (FD4, MW = 4,000 Da; Sigma Aldrich) according to Yuan et al. (2020). FD4 was dissolved in phenol red-free DMEM for a final concentration of 2 mg/mL and 100 μL of the dextran solution were added to the apical side of the inserts. Aliquots of 100 μL were taken from basolateral side after a 2 h incubation at 37°C in the dark. Fluorescence was measured using a fluorescence microplate reader (490 nm excitation/520 nm emission). FD4 transport across the monolayer was quantified using a calibration curve of fluorescence intensity of different FD4 concentrations. Gene expression of tight junction proteins claudin-1 (*Cldn-1*), occludin-1 (*Ocl-1*), and zonula occludens (*Zo-1*) was also determined with Primer PCR SYBR Green Assays as explained above.

### 2.12. Statistical analysis

All data presented are represented as the mean ± standard deviation of at least three replicates. Differences of *p* < 0.05 were considered statistically significant. All statistical analysis was performed in GraphPad Prism V9.4. The survival during digestion was analyzed by a 2-way analysis of variance (ANOVA) and multiple comparison by *post hoc* Tukey test. Adhesion data, qPCR gene expression data, trans-epithelial electrical resistance, and paracellular permeability of FITC-dextran were analyzed with an ordinary one-way ANOVA and *post hoc* Tukey test. EPS production and monosaccharide composition data were analyzed with an unpaired *t*-test.

## 3. Results

### 3.1. Genotypic characterization

**Figure 2:**
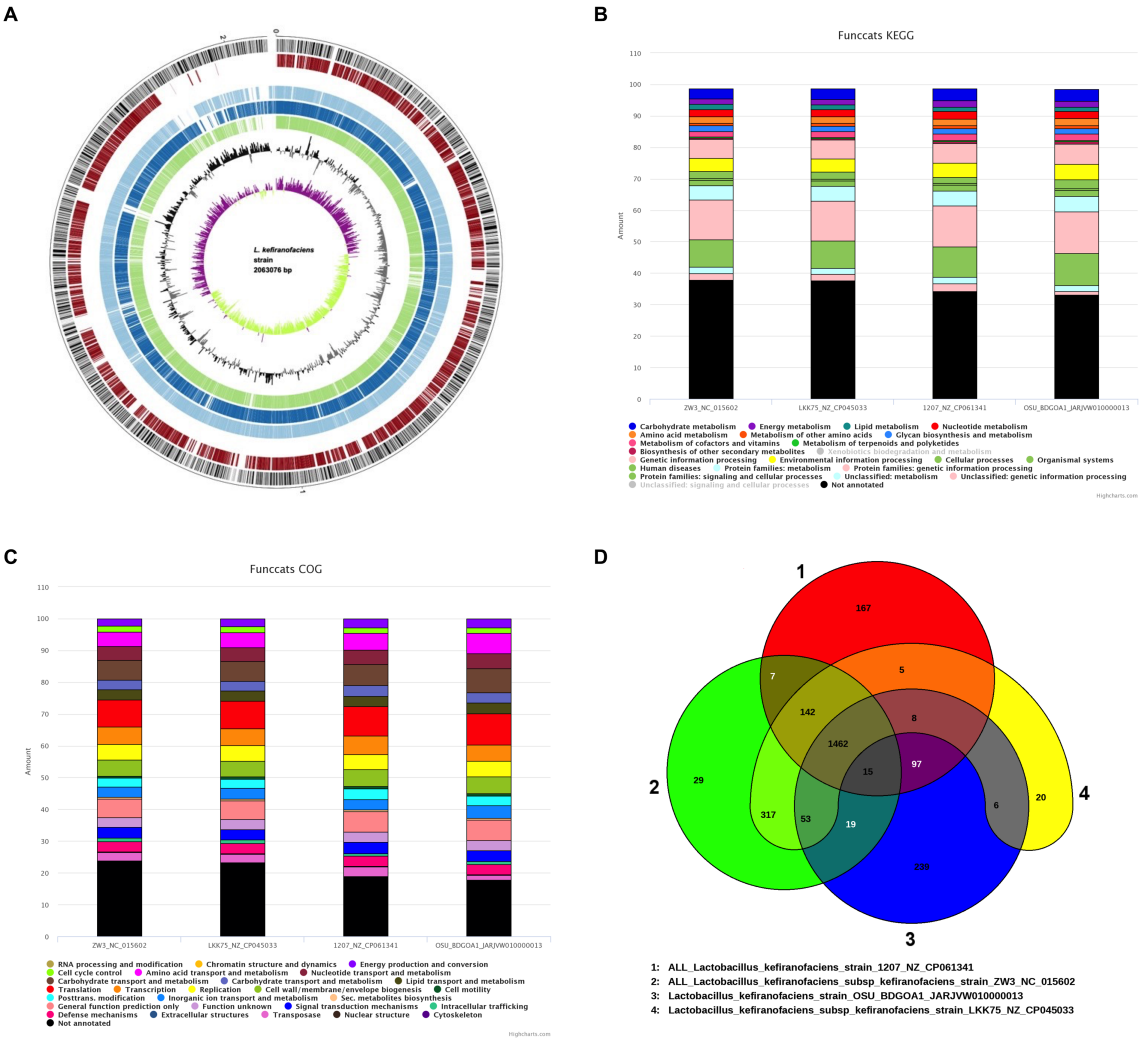
Genomic-based comparison of *Lactobacillus kefiranofaciens* OSU-BDGOA1 and closely related strains of the same species. Circular genome plot **(A)** generated by BioCircos within the EDGAR software comparing four *L. kefiranofaciens* genomes arranged from outer to inner circle as follows: Circles 1–4 showed coding sequences (CDSs) on the forward and reverse strands of *L. kefiranofaciens* OSU-BDGOA1 (reference genome), *L. kefiranofaciens* subsp. *kefiranofaciens* LKK75, *L. kefiranofaciens* 1,207, and *L. kefiranofaciens* subsp. *kefiranofaciens* ZW3, respectively. Circles 5 and 6 represent the RNA features (green) and the GC content. Circle 6 represents the GC skew [(C − G) / (C + G)] curve, above mean GC skew (violet), below mean GC Skew (green). Functional annotation comparison by KEGG **(B)** and COG (clusters of orthologous genes database) **(C)**. Venn diagrams of coding sequences for the four *L. kefiranofaciens* strains evaluated **(D)**.

Conducting gene mining and genome comparison between the strain of interest and related species prior to phenotypic characterization allows for the identification of genetic differences that may be associated with specific traits or functions and therefore guide the experimental design to confirm predictions. The EDGAR comparison revealed 1,575 coding sequences (CDS) shared between *L. kefiranofaciens* OSU-BDGOA1 and *L. kefiranofaciens* 1,207 compared to 1,516 shared CDS with *L. kefiranofaciens* ZW3, and 1,528 shared CDS with *L. kefiranofaciens* LKK75. The average amino acid identity (AAI) between all the tested strains was higher that 99%, indicating a close evolutionary relationship between the given strains. The percentage of conserved proteins (POCP) was higher for the 1,207 strain and the mean nucleotide identity of orthologous genes (FastANI) was higher for the LKK75 strain (Supplementary Figure S1). Furthermore, the gene distribution among the four strains by a Venn diagram revealed that OSU-BDGOA1 has the highest number of unique genes (239) followed by the 1,207 strain (167 unique genes), suggesting that these strains possess greater genomic diversity. Out of the 239 unique genes in OSU-BDGOA1, we found genes encoding polysaccharide synthesis, S-layer, S-layer associated proteins (SLAP), transposases, helveticin J bacteriocin, and genes associated with resistance to acidic conditions. All analyzed *L. kefiranofaciens* strains showed a gene cluster for a bacteriocin III and a bacteriocin immunity protein. The functional categories of the genome were determined using KEGG (Kyoto Encyclopedia of Genes and Genomes) and COG (clusters of orthologous genes database), which enable better understanding of the high-level functions and utilities within the genomes. Figure 2 shows the 10,484 and 8,472 functional gene categories by KEGG and COG, respectively, that were found in all the strains using *L. kefiranofaciens* LKK75 as reference genome.

Genes encoding for an Enterolysin A and a helveticin-J were mined by BAGEL4, while Phage Hunter identified a *Lactobacillus* phage Lfelnf (74% identity) in the OSU-BDGA1 genome that has been reported to infect *Lactobacillus fermentum* (Liu et al., 2015). No genomic islands of virulence factors, resistance genes, or pathogen-associated genes were found in the genome by IslandViewer4. Supplementary Table S2 lists the genes encoding for specific functions that were mined in the genome. The presence of genes associated with bile salt tolerance and resistance to acidic pH, as well as genes associated with EPS production, were identified.

### 3.2. Co-culture increased survival of *Lactobacillus kefiranofaciens* OSU-BDGOA1 during simulated digestion and adhesion to intestinal cells

**Figure 3:**
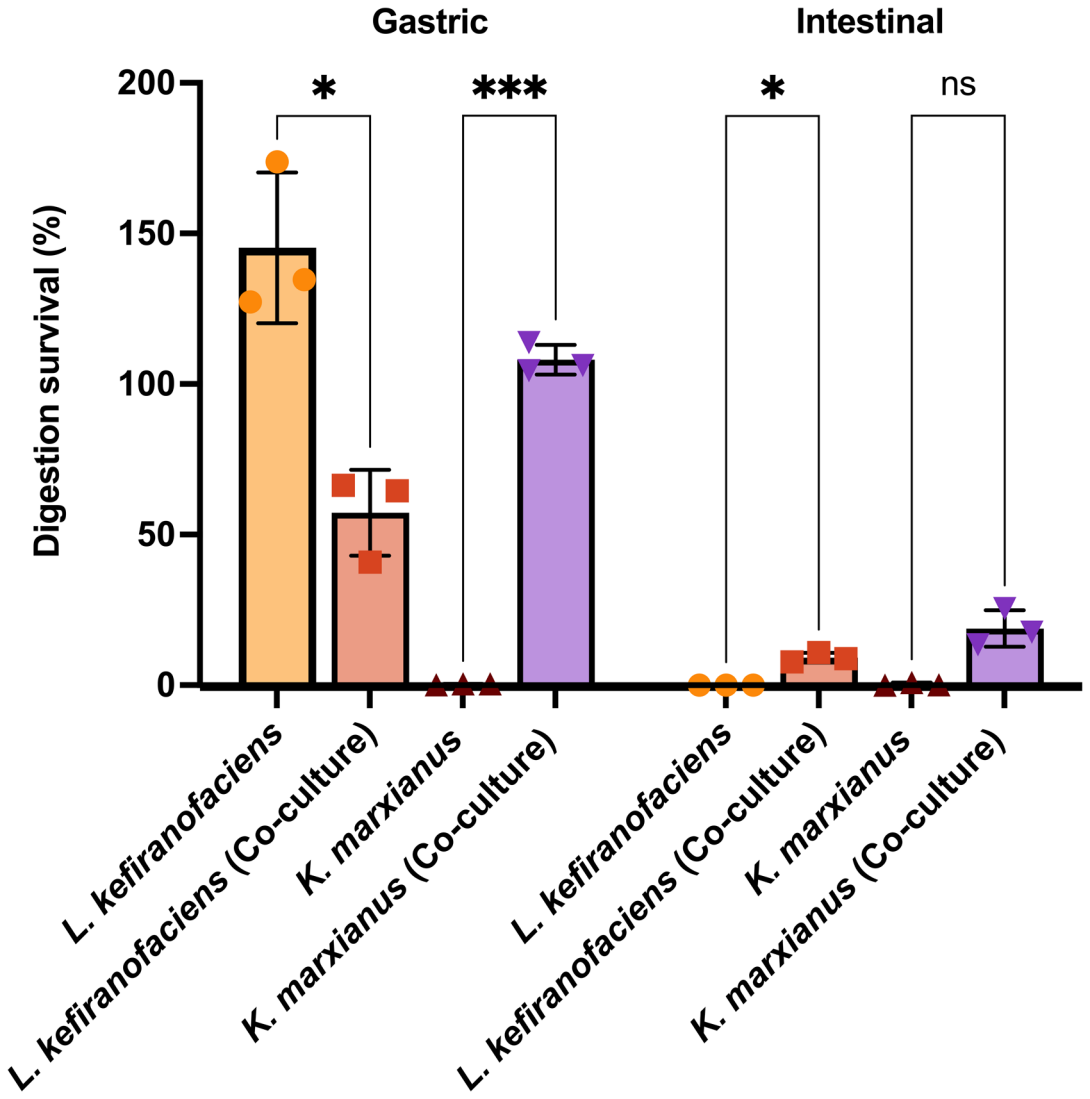
Survival (%) of *L. kefiranofaciens* and *K. marxianus* in mono- and co-culture across the gastric and intestinal phases using the INFOGEST static simulated digestion model. Statistical difference between treatments is denoted as *p < 0.05, **p  < 0.01, ***p < 0.0005 by 2-way ANOVA with *post hoc* Tukey test.

**Figure 4:**
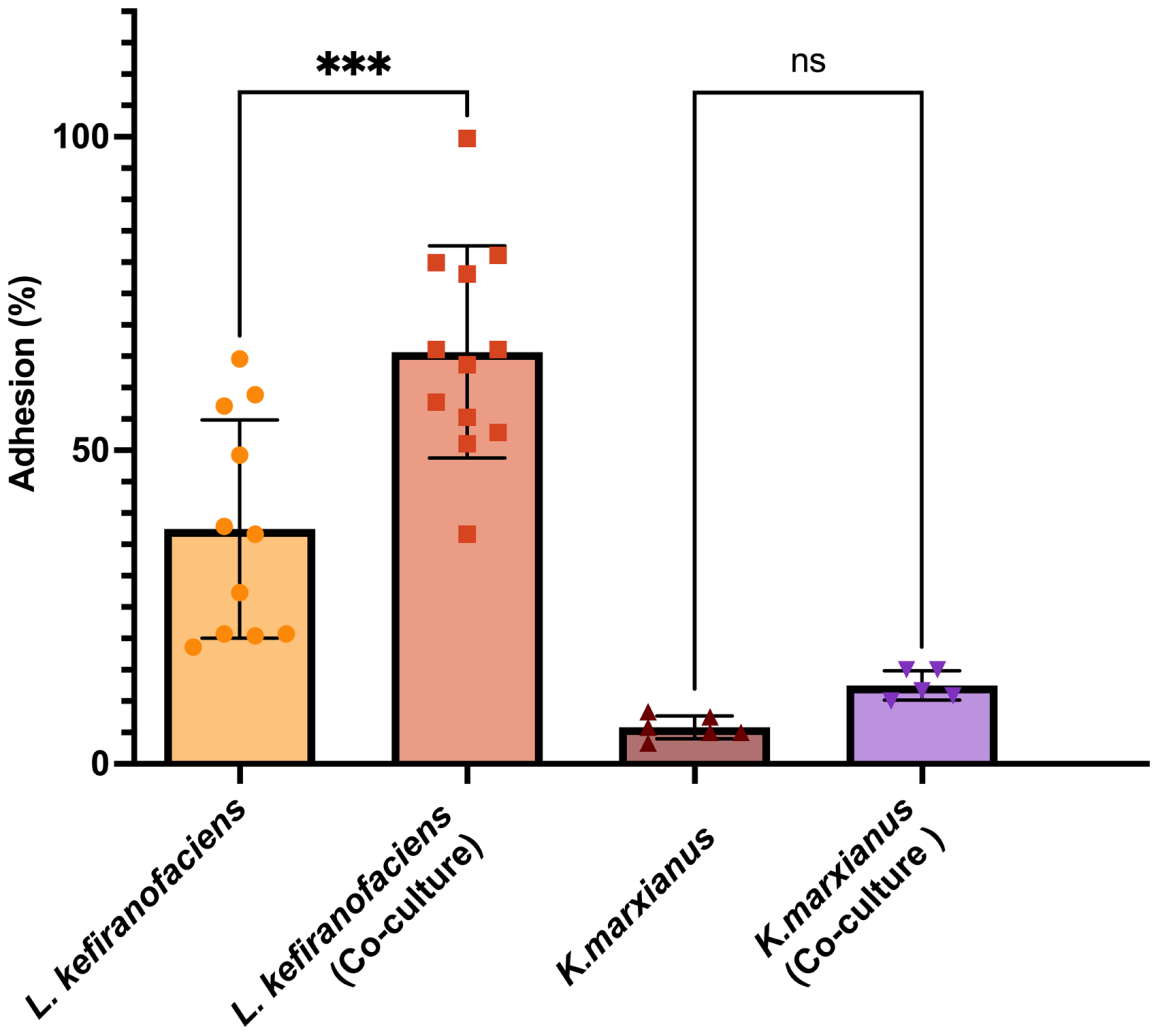
Adhesion (%) of *L. kefiranofaciens* and *K. marxianus* in mono- and co-culture to Caco-2 intestinal cells. Values represent the mean ± SD of at least three replicates. Statistical difference denoted as ***p < 0.0005 by one-way ANOVA with post hoc Tukey test.

To determine whether the functional characteristics found in the genome of *L. kefiranofaciens* OSU-BDGOA1 are associated with the expressed phenotype and whether they are influenced by co-culturing with *K. marxianus*, we screened for several key probiotic properties, including survival during digestive stress and adhesion to intestinal cell culture. During simulated digestion, the survival of *L. kefiranofaciens* significantly increased from 0.003 to 9.14% (*p* < 0.05) at the end of the intestinal phase when co-cultured with *K. marxianus* bdgo-ym6. For *K. marxianus*, the survival was significantly greater in co-culture in the gastric phase (109% vs. 0.31%); however, at the end of the intestinal phase, the difference between co-culture and monoculture was not significant (18.8 and 0.35%, respectively) (Figure 3). The effect of the co-culture in modifying the adhesion phenomenon of these microorganisms to intestinal cells was assessed with the Caco-2 cell culture model using a 4 h incubation to simulate gastrointestinal transit time. The co-culture condition enhanced adhesion of *L. kefiranofaciens* compared to the monoculture (65.68, and 37.47%, respectively). This result represents a 1.7-fold increase in adhesion to intestinal cells. However, the adhesion of *K. marxianus* was not enhanced, with a 12.5% adhesion in the co-culture to 5.8% in the monoculture condition (Figure 4).

### 3.3. EPS production increased in the co-culture using mCGB in a time-dependent manner

**Figure 5:**
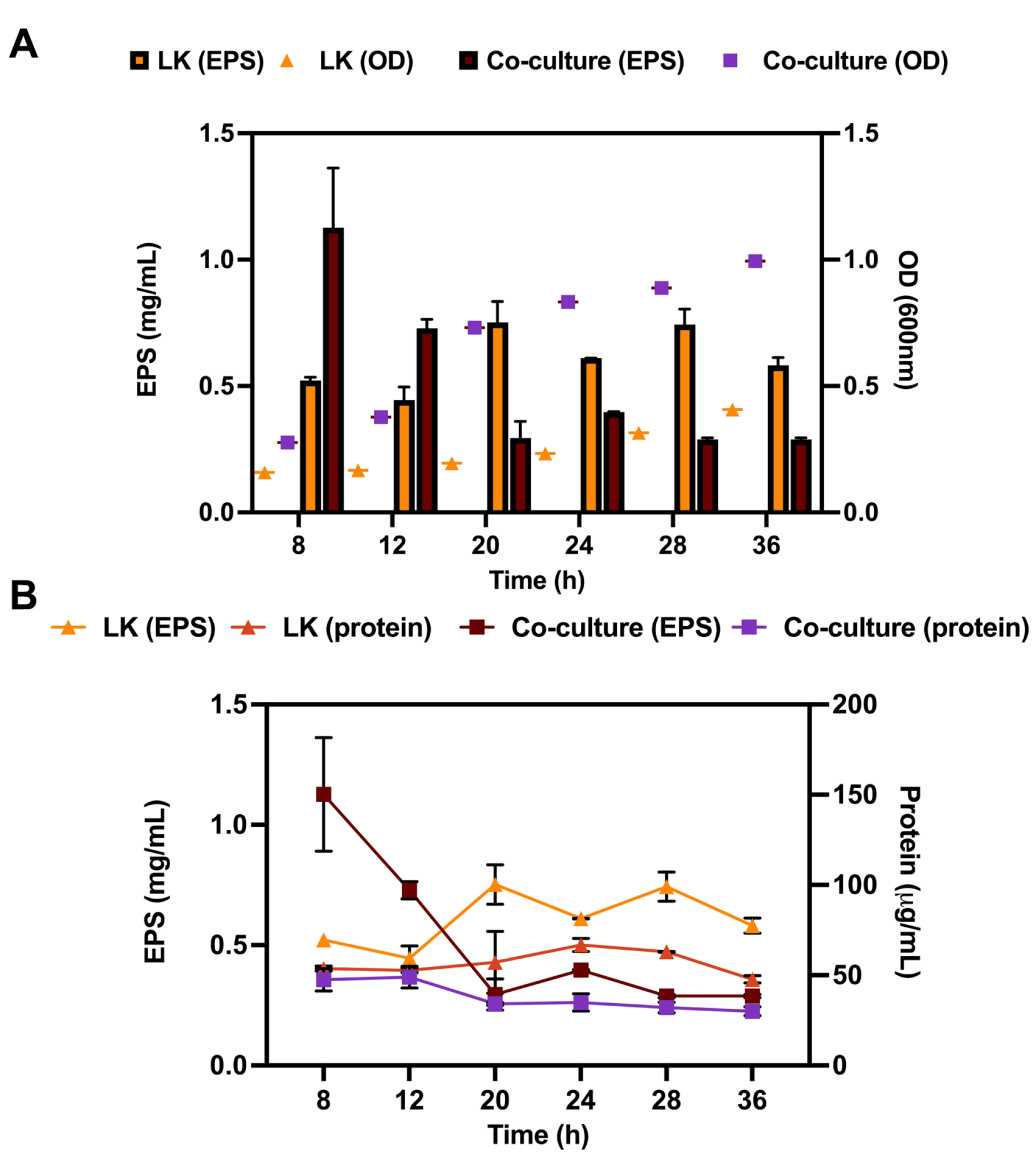
Exopolysaccharide production curves (left y-axis) compared to protein quantification curves (right y-axis) of *L. kefiranofaciens* in mono and co-culture with *K. marxianus* during 36 h at 30°C **(A)**. Exopolysaccharide production curves (left y-axis) compared to growth curve (right y-axis) of *L. kefiranofaciens* in mono and co-culture with *K. marxianus* during 36 h at 30°C **(B)**. Values represent the mean ± SD of at least three replicates.

EPS production in the co-culture and monoculture was determined throughout microbial growth in mCGB. The growth of the microorganisms was determined by plate count on selective media and the EPS production was normalized for bacterium number. EPS production in co-culture peaked at 8 h in the exponential phase and was higher than in the *L. kefiranofaciens* monoculture (0.73 and 0.44 mg/mL, respectively). However, in the late exponential phase (*t* = 12 h) the EPS production was significantly reduced in the co-culture compared to the monoculture, with values of 0.4 mg/mL for co-culture to 0.60 mg/mL for the monoculture (*p* < 0.05) (Figure 5A). The decreasing trend in EPS production for the co-culture was maintained for the remaining hours of the fermentation, although after 20 h no significant differences were observed between both conditions. Since carbohydrates from peptidoglycan and glycoproteins may be co-extracted and contribute to the EPS quantification, the protein content was monitored and compared to the EPS production at each time point. The protein quantification did not significantly change during microbial growth under any of the conditions (Figure 5B).

**Figure 6:**
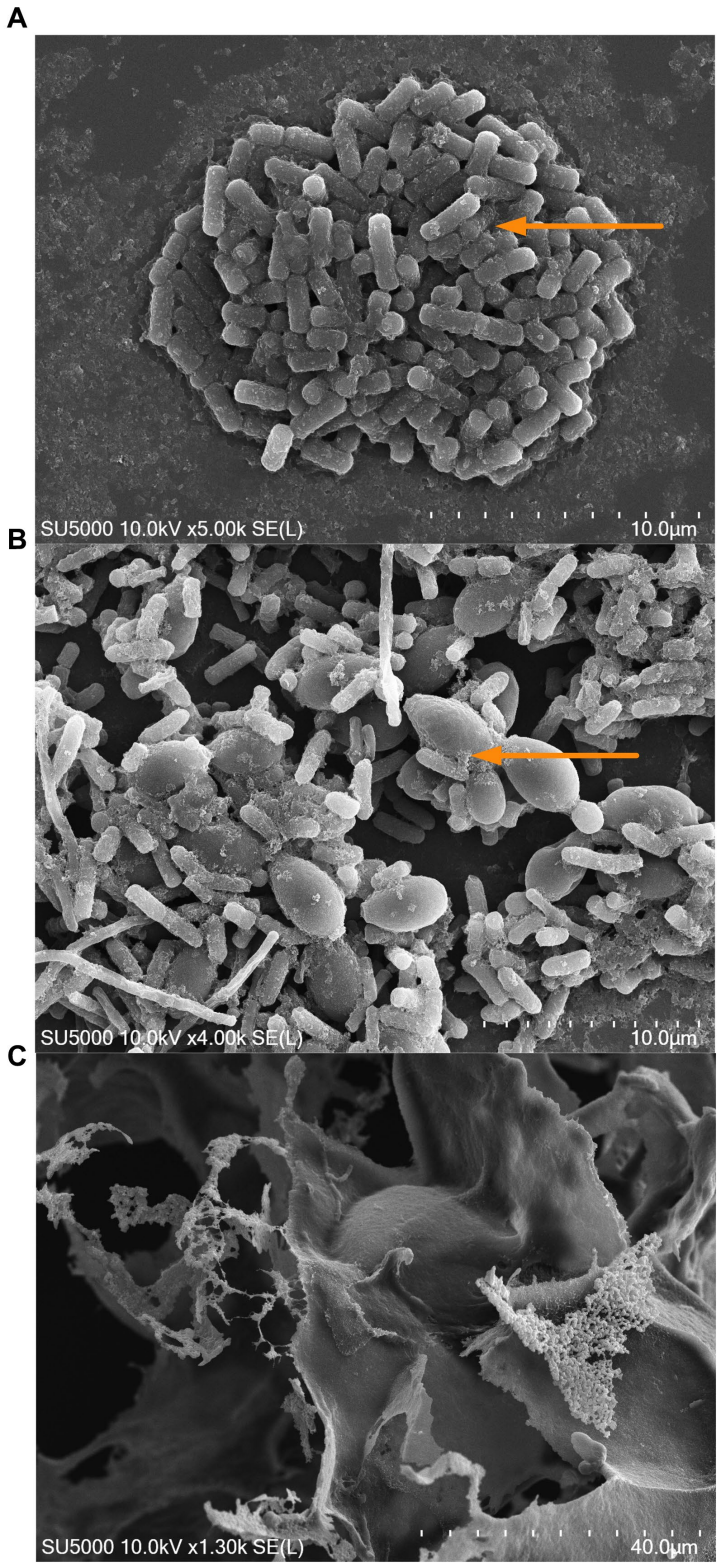
Scanning electron microscopy (SEM) images of *L. kefiranofaciens* monoculture **(A)**, *L. kefiranofaciens* and *K. marxianus* co-culture **(B)**, and exopolysaccharide produced by *L. kefiranofaciens* **(C)**. Arrow indicates the exopolysaccharide network observed in the mono- and co-culture.

As a secondary approach, and to determine the monosaccharide composition of the EPS produced in the co-culture and monoculture at the stationary phase (16 h), we used HPLC-CAD. Carbohydrates extracted from both the co-culture and monoculture displayed similar neutral sugar profiles comprising of D-Glucose (D-Glu), D-Galactose (D-Gal), and D-Mannose (D-Man) (Table 1). Total sugars were higher in the co-culture compared to the monoculture, consisting of 139.1 and 81.9 g/100 g freeze-dried powder, respectively (*p* < 0.05). The ratio of D-Glu to D-Gal was significantly different in the monoculture condition as compared to the co-culture (1:0.9 and 1:1.5, respectively). The results are in alignment with the observed increased in the EPS production at the early and late stationary phases of the microbial growth (Figure 5A). The visualization of the mono and co-culture via SEM revealed co-aggregation between bacteria and yeast cells in microbial clusters surrounded by EPS layers (Figures 6A,B). The EPS may act as a protective layer and provide mechanical stability to the consortium by serving as a network to maintain a close association between both species. This association may facilitate cross-talk between the cells and activation of genes that allow the co-culture to survive stressful environmental conditions (Kapoore et al., 2022).

**Table 1:** Monosaccharide composition of the exopolysaccharide (EPS) produced by *Lactobacillus kefiranofaciens* (LK) monoculture and co-culture (LK-KM) after 16 h of incubation at 30°C.

Treatment	Total Sugars (g/100 g freeze-dried powder)^*^	D-Glu g/100 g	D-Gal g/100	D-Man g/100	Ratio Glu:Gal:Man
LK	81.9 ± 3.7^b^	25.3 ± 8.5^b^	23.3 ± 5.1^b^	33.3 ± 1.1^b^	1:0.9:1.3
LK + KM	139.1 ± 2.5^a^	33.6 ± 2.2^a^	50.4 ± 6.2^a^	55.1 ± 6.7^a^	1:1.5:1.6

^*^Values correspond to the mean ± SD. Means without common letter between treatments are significantly different (*p* < 0.05), Student’s *t*-test.

### 3.4. Antibacterial activity in cell-free supernatant comes from a proteinaceous compound

To determine whether the co-culture exerted an impact on the antibacterial activity against indicator strains, and to gain insight into the nature of the inhibitory compounds, we treated the CFS of *L. kefiranofaciens* OSU-BDGOA1 in mono- and co-culture with catalase and trypsin. The antibacterial effects depicted by the CFS against *Escherichia coli* ATCC 25922 and *Listeria innocua* ATCC 51742 were not affected by the co-culture conditions (Table 2 Supplementary Figure 2). Conversely, the antibacterial effect was not influenced by catalase treatment, indicating that hydrogen peroxide could not be responsible for the observed effect. However, when the CFS was treated with trypsin, the antibacterial effects against both microorganisms were completely depleted. The heat control also retained the effect, which suggests that the compound responsible for the antibacterial activity is likely proteinaceous in nature, and that upon proteolysis it loses its effect.

**Table 2:** Antibacterial activity of cell-free supernatants (CFS) from *L. kefiranofaciens* (LK) in mono- and co-culture with *K*. *marxianus* (LK-KM) against indicator strains by the agar well diffusion assay^*^.

Treatment	*E. coli* ATCC 25922		*L. innocua* ATCC 51742	
	LK	LK-KM	LK	LK-KM
CFS	16.05 ± 0.07	14.15 ± 1.63	14.15 ± 0.49	14.05 ± 0.71
CFS + catalase	12.4 ± 0.14	12.2 ± 0.28	13.65 ± 0.21	12.75 ± 1.06
CFS + trypsin	ND	ND	ND	ND
CFS heat control	13.25 ± 0.35	13.5 ± 0.71	12.75 ± 0.35	12.0 ± 0.0

^*^Values correspond to the mean ± SD of the diameter of the inhibition halo (mm). ND: not detected antibacterial activity.

### 3.5. The monoculture and co-culture of *Lactobacillus kefiranofaciens* OSU-BDGOA1 prevents and ameliorates *Kluyveromyces marxianus* monoculture exacerbation of HKSC-induced inflammation in Caco-2 cells

**Figure 7:**
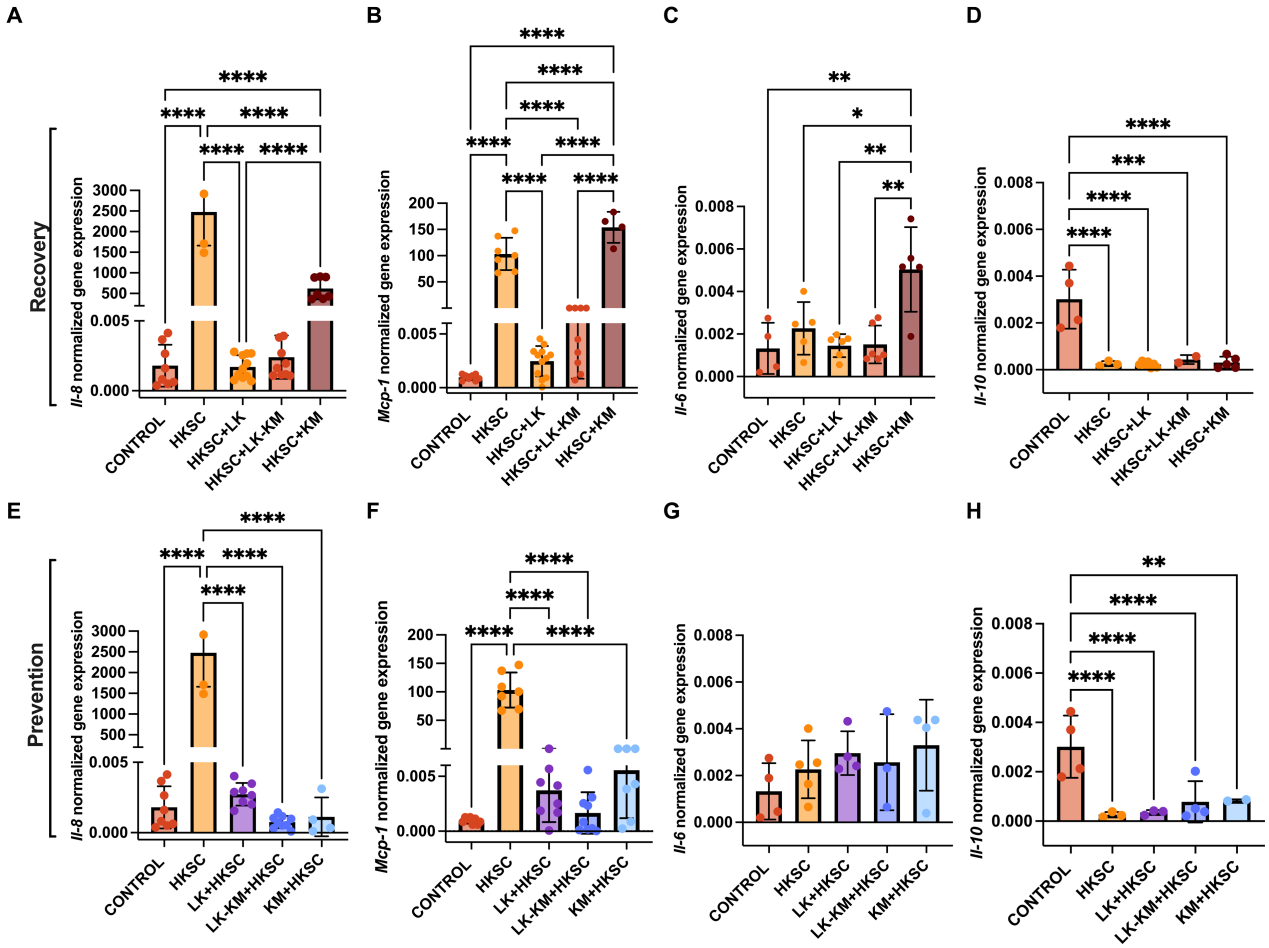
Effect of co-culture (LK-KM) and monocultures (LK, KM) on the gene expression of cytokines (*Il-8, Mcp-1, Il-6, Il-10*) for recovery **(A–D)** and prevention **(E–H)** of HKSC-induced inflammation in Caco-2 cells. Values represent the mean ± SD of at least three replicates. Statistical difference denoted as *p < 0.05, **p < 0.01, ****p < 0.0001 by one-way ANOVA with *post hoc* Tukey test.

The potential protective effect of the co-culture and monocultures at preventing and resolving HKSC-induced inflammation was assessed in Caco-2 cells. Treatment with the co-culture (LK-KM) and *L. kefiranofaciens* monoculture (LK) in both the recovery and prevention groups resulted in significant downregulation of the pro-inflammatory cytokines *Il-8* and *Mcp-1* gene expression, with values similar to those of the untreated cells control (*p* < 0.05) (Figures 7A,B,E,F). Interestingly, the monoculture of *K. marxianus* (KM) in the recovery group significantly upregulated the expression of *Il-8*, *Mcp-1*, and *Il-6* to levels similar to the HKSC. *Il-6* expression levels in the prevention group were not significantly affected by any of the treatments (*p* < 0.05) (Figure 7G), whereas in the recovery group the KM monoculture significantly induced *Il-6* expression as compared to the HKSC and untreated cells control (Figure 7C). *Il-10* expression was not induced by any of the treatments as compared to the untreated cells control.

### 3.6. *Lactobacillus kefiranofaciens* OSU-BDGOA1 in mono- and co-culture protects against HKSC-induced disruption of intestinal barrier function by regulating tight junction proteins expression

**Figure 8:**
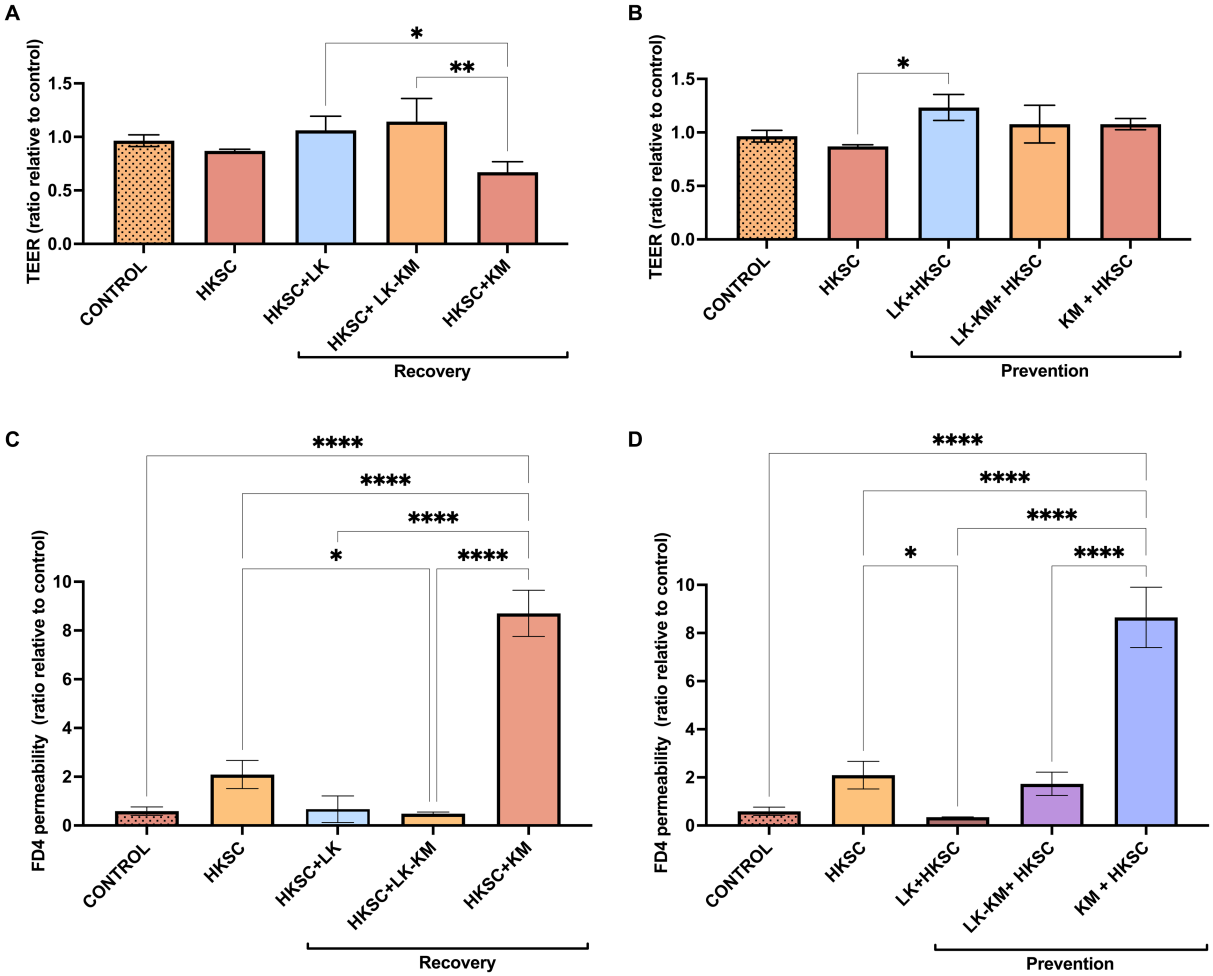
Effect of co-culture (LK-KM) and monocultures (LK, KM) on trans-epithelial electrical resistance (TEER) and paracellular permeability of FITC-dextran (FD4) for recovery **(A,C)** and prevention **(B,D)** of HKSC-induced barrier damage. Statistical difference denoted as *p < 0.05, **p < 0.01,****p < 0.0001 by one-way ANOVA with *post hoc* Tukey test.

In the recovery group, treatment with HKSC led to a 14% decrease in TEER in Caco-2 cells. However, when treated with *K. marxianus* monoculture (HKSC+KM), TEER was markedly more decreased, with a 35% TEER reduction compared to the untreated cells control. In the prevention group, treatment with LK in monoculture significantly increased TEER compared to the HKSC (*p* < 0.05) (Figures 8A,B).

In the paracellular permeability assay, the FD4 concentration across the membrane increased by 3.5-fold in cells treated with HKSC, and remarkably by 14.6-fold in the *K. marxianus* prevention group (KM + HKSC), and by 14.7-fold in the *K. marxianus* recovery group (HKSC+KM). These results suggest a significant reduction in the membrane barrier integrity in these treatments by loss of Caco-2 cell junctions. Treatment with *L. kefiranofaciens* co-culture in the recovery group (HKSC+LK-KM) and with monoculture in the prevention group (LK + HKSC) significantly reduced FD4 paracellular transport compared to HKSC (*p* < 0.05). Furthermore, in both the recovery and prevention groups *L. kefiranofaciens* mono- and co-culture showed values similar to those of the untreated control cells, indicating conservation of Caco-2 cell junctions (Figures 8C,D).

**Figure 9:**
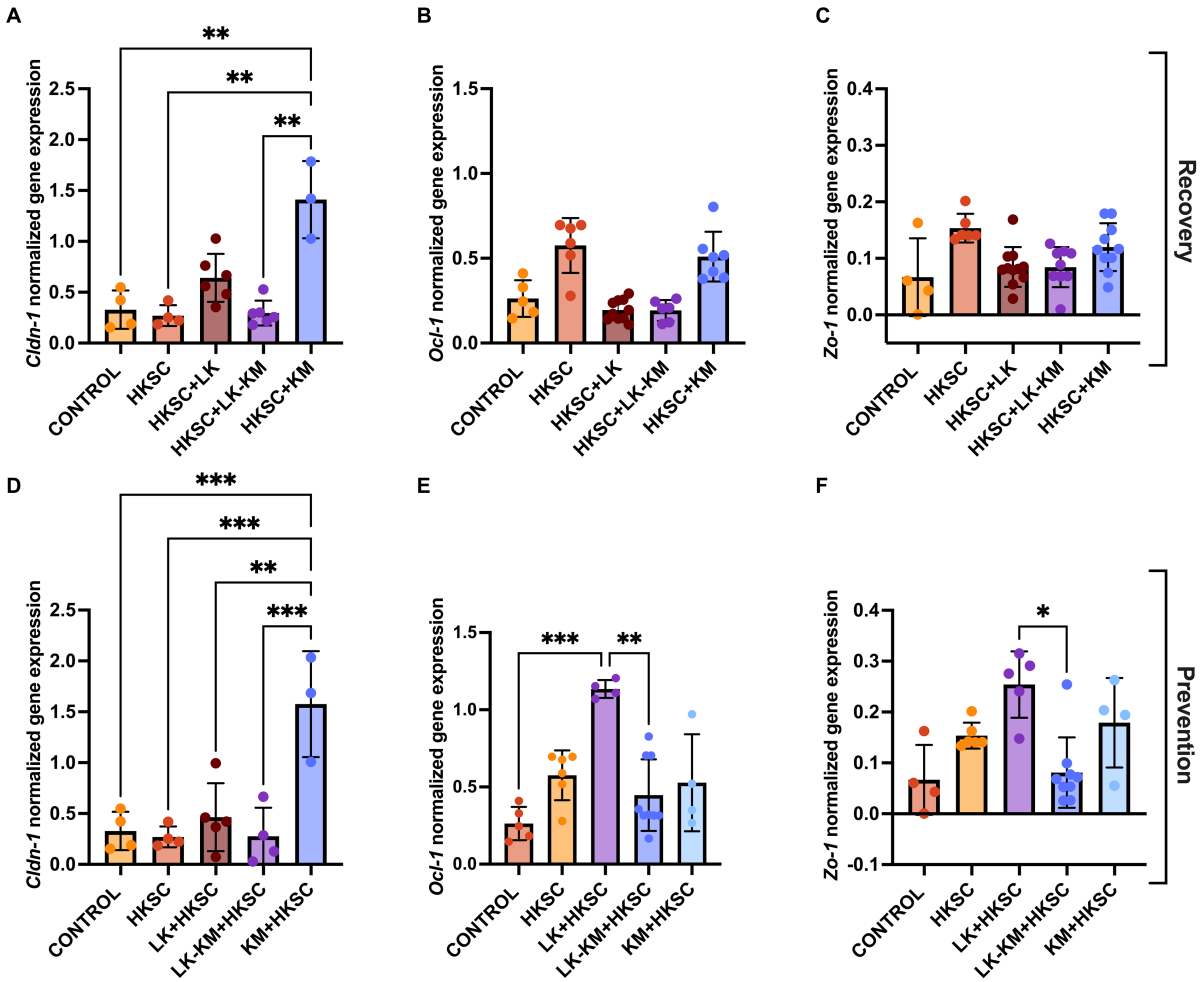
Effect of co-culture (LK-KM) and monocultures (LK, KM) on the expression of tight junctions (*Cldn-1-1, Ocl-1, Zo-1*) for recovery **(A–C)** and prevention **(D–F)** of HKSC-induced barrier damage in Caco-2 cells. Values represent the mean ± SD of at least three replicates. Statistical difference denoted as *p  < 0.05, **p < 0.01,***p < 0.0005 by one-way ANOVA with *post hoc* Tukey test.

To determine if the disruption of the intestinal barrier was associated with altered gene expression of tight junctions, we analyzed the expression of *Cldn-1*, *Ocl-1*, and *Zo-1*. In both the recovery and prevention groups, treatment with HKSC did not reduce the expression of the three tight junction proteins as compared to the untreated cells (Figure 9). In the recovery group, the gene expression of *Ocl-1* and *Zo-1* was not affected by any of the treatments, including HKSC (Figures 9B,C). However, there was a significant induction in *Cldn-1* expression in the *K. marxianus* monoculture in both the recovery and the prevention group (*p* < 0.05) (Figures 9A,D). Furthermore, in the prevention group, treatment with *L. kefiranofaciens* monoculture (LK + HKSC) showed significantly higher expression levels of *Ocl-1* and *Zo-1* as compared to the co-culture (LK-KM + HKSC) (*p* < 0.05) (Figures 9E,F).

## 4. Discussion

In the present study, the effect of co-culturing *L. kefiranofaciens* OSU-BDGOA1 with *K. marxianus* bdgo-ym6 was compared to the respective monocultures. The survival of probiotic strains throughout gastrointestinal transit and their adhesive ability to epithelial cells are key factors that determine their beneficial effects. Survival of various *L. kefiranofaciens* strains isolated from kefir and kefir grains in gastrointestinal conditions has been reported by other authors. Kim et al. (2017) reported that two *L. kefiranofaciens* strains isolated from kefir displayed high survival and growth (final bacteria growth exceeded the initial count) during gastrointestinal conditions. In our study, *L. kefiranofaciens* OSU-BDGOA1 in monoculture exceeded the initial counts at the gastric phase; however, at the end of the intestinal phase, the survival was significantly reduced. Evidence of survival capacity in gastrointestinal conditions was found in the genome of *L. kefiranofaciens* OSU-BDGOA1 with genes encoding for resistance to acid and bile salt conditions. Survival of *K. marxianus* in gastrointestinal conditions had been previously reported by others, Fadda et al. (2017) showed that four strains isolated from Fiore Sardo cheese showed higher survival as compared to the probiotic commercial yeast *Saccharomyces boulardii* CODEX SB1with survival rates from 83 to 100%, and adhesion to Caco-2 cells ranging from 4 to 68%. Recently, another study evaluated the survival of five *K. marxianus* strains isolated from kefir in different gastrointestinal environments and compared it to the survival depicted by *S. boulardii* MYA-796. The authors observed not only survival but yeast growth from 1.1 to 11.2 fold (Youn et al., 2022). However, comparing survival in gastrointestinal conditions among different studies is challenging due to the different protocols and conditions that try to simulate the complex gastrointestinal environment (Brodkorb et al., 2019). The highest survival in the present study showed by the co-culture of *L. kefiranofaciens* OSU-BDGOA1 and *K. marxianus* bdgo-ym6 could be associated with the increased production of EPS during the late exponential and early stationary phases of co-culture growth. It has been shown that EPS can enhance the viability of probiotics in the gastrointestinal tract by acting as a protective layer during gastrointestinal transit (Lebeer et al., 2011).

In this study, the observed decrease in EPS in the co-culture after 12 h may be attributed to the limited availability of nutrients in the medium. To minimize the interference of other medium components containing reducing sugars, mCGB was used, which allowed specific quantification of bacterial EPS. However, the removal of yeast extract and beef extract had an impact on the growth of both mono- and co-cultures. Under these restricted nutrient conditions, it is possible that the yeast and bacterium are utilizing the EPS as a fermentable nutrient source to support their growth. This is considered a potential benefit of the EPS in the gut, as they can serve as a fermentable substrate for the gut microbiota (Oerlemans et al., 2021). The monosaccharide composition of the EPS produced by *L. kefiranofaciens* has been previously described as a glucogalactan with a glucose and galactose ratio of approximately 1:1 (Exarhopoulos et al., 2018). While co-culturing *L. kefiranofaciens* with non-EPS producing yogurt bacteria has been shown to alter the EPS composition by adding additional sugars such as xylose and arabinose (Ahmed et al., 2013), no changes in the EPS composition between mono- and co-culture were observed based on the limited neutral sugars we analyzed by HPLC-CAD. However, potential changes in other neutral sugars may have occurred.

Previously, kefiran production by *L. kefiranofaciens* was enhanced by a co-culture system of yeast-LAB, the authors observed that the yeast prevents lactic acid accumulation allowing LAB growth and therefore increasing EPS production (Tada et al., 2007). Previous research reported that the ideal pH for maximum kefiran production lies between 5 and 6 (Cheirsilp and Radchabut, 2011; Zajšek et al., 2013). In our study, the pH in the co-culture at 12 h was 5.22, compared to 4.28 in the monoculture (Supplementary Table S3), supporting the important role of the yeast in enhancing EPS production by regulating media pH. Additionally, yeast can support the growth of LAB through production of diffusible growth-promoting factors, with amino acids being particularly important since most of LAB are auxotrophic for important amino acids (Ponomarova et al., 2017; Liu et al., 2022). In particular, *S. cerevisiae* EC118 has been shown to supply alanine to *L. delbrueckii* when cultivated in co-culture, leading to increased viability of *Lactobacillus rhamnosus* HN001 and *Lacticaseibacillus paracasei* H9 under gastrointestinal conditions and improved adhesion to intestinal cells (Lim et al., 2015).

The SEM images of the co-culture also showed cell–cell interactions between bacteria and yeast cells that could be attributed to the potential increase in survival during gastrointestinal conditions. Previous studies have reported that direct cell contact between *L. rhamnosus* and *S. cerevisiae* in co-culture can increase viability through cell wall components and metabolites (Xu et al., 2021). Furthermore, yeast cell wall components can also play a significant role in increasing adhesion to intestinal cells. The co-aggregation of *Lactobacillus* spp. and *S. cerevisiae* via mannose-specific adhesin (Msa) and surface layer proteins (S-layer proteins) can mediate adhesion to intestinal cells (Xu et al., 2021). In a study by Wang et al. (2023), increased adhesion to Caco-2 cells of kefir grain isolates *Lactobacillus helveticus* SNA12 and *K. marxianus* GY1 in co-culture was attributed to the presence of proteins and polysaccharides in the yeast cell wall. Furthermore, co-aggregation has been shown to induce overproduction of EPS by *L. kefiranofaciens* in co-culture with *S. cerevisiae* (Benjamas et al., 2003).

Genome mining of *L. kefiranofaciens* OSU-BDGOA1 for genes associated with bacteriocin production revealed the presence of genes that encode class III bacteriocins; Enterolysin- A, a cell wall degrading bacteriocin with broad spectrum activity against Gram-positive and Gram-negative bacteria (Nilsen et al., 2003) and helveticin J, a bacteriocin with a narrow antibacterial spectrum against Gram-positive bacteria including *Listeria monocytogenes* (Joerger and Klaenhammer, 1986), and that has been previously found in other *L. kefiranofaciens* strains (Xing et al., 2017; Georgalaki et al., 2021). However, we cannot directly attribute the observed *in vitro* antibacterial activity to the bacteriocins mined from the genome. The production of bacteriocins *in vivo* is regulated by complex signaling pathways that may not be expressed in experimental conditions. Additionally, post-translational modifications can affect the stability and activity of the bacteriocins, leading to different activities. Finally, other proteins/peptides that are not of bacteriocin nature could be associated with the observed activity (Huang et al., 2021).

In this study, we used an inflammation model using HKSC in Caco-2 epithelial cells to study the immune responses of the co-culture and respective monocultures. Previous research reported that some commonly used inflammation inducers such as bacterial lipopolysaccharide (LPS) did not induce an IL-8 response in *in vitro* intestinal cell models including Caco-2 cells, indicating that the Toll-like receptors (TLRs) that recognize these pathogen-associated molecular patterns (PAMPs) are either not present or not functional in these models. However, flagellin from *Salmonella* Typhimurium significantly increased IL-8 in the apical side of a Caco-2 cells, suggesting that TLR5, which recognizes flagellin, is present and functional in this cell culture model (Grouls et al., 2022). Carasi et al. (2014) induced inflammation in Caco-2 cells by using flagellin from *Salmonella* Typhimurium (FliC) to study the immune response of different *Lentilactobacillus kefiri* strains after 6 h of treatment. However, utilization of HKSC is a cost-effective and less hazardous way to study inflammation in cell-culture models. As Kalupahana et al. (2005) reported, the viability of *Salmonella* Typhimurium is not essential for the activation of the inflammatory pathways as a 2 h stimulation of HKSC induced inflammation in macrophage-like and dendritic cell lines. Activation of the immune response after *Salmonella* invasion is mainly associated with stimulation of CCL20 chemokine by flagellin, which triggers dendritic cell chemotaxis and secretion of IL-8 (Sierro et al., 2001).

Previous studies have shown the *in vitro* anti-inflammatory effects and membrane barrier properties of *L. kefiranofaciens* and *K. marxianus* strains in monoculture using different models of inflammation (Chen et al., 2013; Romanin et al., 2016; Smith et al., 2016; Xing et al., 2017). However, there is limited research on the potential regulation of inflammation by a co-culture of these microorganisms. Recently, kefir grain isolates *L. kefiranofaciens* and *S. cerevisiae* in mono- and co-culture enhanced the gut barrier and decreased the expression of pro-inflammatory cytokines in a colon inflammation and colorectal carcinogenesis mouse model (Zeng et al., 2022). In the present study, we observed a decrease in the expression of pro-inflammatory cytokines *Il-8* and *Mcp-1* by *L. kefiranofaciens* in both mono- and co-culture with *K. marxianus* as a preventative and restorative treatment as compared to the expression induced by HKSC. These findings could potentially help the host immune response upon an inflammatory event. Interestingly, treatment with *K. marxianus* monoculture in the recovery group showed markedly higher expression of *Il-8* and *Mcp-1*. Yeast cell wall components can have beneficial or adverse effects in a strain-dependent manner on intestinal inflammation upon recognition by TLRs. Furthermore, fungal microbiota are important regulators of gut dysbiosis and their interaction with bacteria in the gut are important regulators of disease (Hall and Noverr, 2017). Cell wall β-glucans of various *S. cerevisiae* strains showed exacerbation of the inflammatory response in a mice model, whereas *S. boulardii* CNC I-3799 down-regulated the pro-inflammatory response (Jawhara et al., 2012). Although the mechanisms by which *K. marxianus* bdgo-ym6 seems to exacerbate the inflammation induced by HKSC remain to be elucidated, the understanding of these complex interactions and the role of the yeast cell wall components on the inflammation mechanisms may provide a rational for future studies in yeast-LAB co-cultures. While in this study only the gene expression of the cytokines was evaluated, a similar study reported a correlation between gene expression and protein levels of cytokines (Zeng et al., 2022).

The intestinal epithelial cells and cell connections are the first barrier of defense against invasion of pathogenic microorganisms and toxic compounds, and its disruption implies entrance to the gut lumen and ultimately circulatory and tissue invasion (Slifer and Blikslager, 2020). Tight junctions (TJ) composed of transmembrane proteins such as claudins, occludin (OCL), and zonula occludens (ZO) play a crucial role in regulating paracellular pathway transport and in maintaining intestinal barrier integrity (Li et al., 2021). TJ play a key role in the proper functioning of the epithelial barrier and exposure to foreign substances can compromise their function, causing a decrease in epithelial resistance and increase in paracellular permeability. This disruption subsequently leads to activation of inflammatory pathways and elevated levels of pro-inflammatory cytokines (Bhat et al., 2019). In the present study, the expression levels of *claudin-1*, *Ocl-1*, and *Zo-1* were evaluated in the different treatment groups. Overall, the expression levels were not affected by HKSC as compared to the untreated control cells. *Salmonella* Typhimurium disrupts TJ by type 3 secretion system (T3SS) protein complexes that translocate specific proteins directly in the host cells (Anderson and Kendall, 2017). In the case of HKSC, this activation was not achieved, however, flagellin has been shown to disrupt membrane barrier by disruption of TJ organization and structure, which would explain the reduced TEER and increased FD4 permeability but negligible effect on TJ gene expression. Varying effects of different probiotic strains on the expression levels of TJ proteins have been reported. Previous exposure to *Lactobacillus acidophilus*, *L. rhamnosus*, and *L. casei* to HT29-MTX intestinal cells had a protective effect against *Salmonella enterica* ser. Javiana invasion and cell damage (Burkholder et al., 2019), while treatment with *L. plantarum* with prior challenge by enterotoxigenic *Escherichia coli* (ETEC) increased expression of *Cldn-1*, *Ocl-1*, and *Zo-1* and protected against membrane barrier disruption in IPEC-J2 cells (Wang J. et al., 2018). Zeng et al. (2022) reported that the oral administration of *L. kefiranofaciens* JKSP109 and *S. cerevisiae* JKSP39 in mono- and co-culture alleviated the effects of dextran sulfate sodium (DSS)-induced colorectal cancer in mice by increasing the expression and protein levels of tight junction proteins to various degrees. In the present study, *Ocl-1* and *Zo-1* expression was upregulated by *L. kefiranofaciens* monoculture as a preventative treatment, which may be associated with higher TEER and reduced FD4 permeability observed by this treatment. Importantly, the maintenance of membrane barrier could be also mediated by the postbiotic effects of EPS, as it has been reported that EPS protect against membrane disruption by upregulation of tight junction protein expression (Oerlemans et al., 2021).

Of notable interest is the increase in *Cldn-1* gene expression in the *K. marxianus* monoculture in both the recovery and prevention groups, along with the concomitant increase in FD4 permeability. Similarly, Poritz et al. (2011) reported an increase in *Cldn-1* expression while *Ocl-1* expression and TEER levels decreased during an inflammation induction with TNF-α in IEC-18 cells. These results are in alignment with the hypothesis that the increased expression of TJ proteins may actually be a protective response provoked by initial degradation of the TJs as suggested by Rose et al. (2021). These authors observed that treatment with *L. plantarum* in Caco-2 cells resulted in increased transcription of genes involved in tight junction disassembly and occludin-1 degradation. Therefore, a clear association between expression of TJ proteins and membrane barrier upon probiotic treatments remains unclear.

## 5. Conclusion

The complex microbial interactions that take place within co-cultures and the complex dynamics between bacteria and yeast play a significant role in the fermentation of traditional milk kefir. Understanding the interactions between *L. kefiranofaciens* and *K. marxianus* in co-culture, can provide valuable insights for development of a standardized commercial product with active core kefir microorganisms. Collectively the data presented in this study shows that co-culturing *L. kefiranofaciens* OSU-BDGOA1 with *K. marxianus* bdgo-ym6 in MRS medium enhances the probiotic potential of these strains, as evidenced by the increased survival during gastrointestinal conditions and enhanced adhesion to epithelial cells. These results, along with the modulation of the immune response in intestinal cells, highlights the opportunity to use co-cultures of yeast and LAB in novel fermented functional products that support human health. However, further research is required with an *in vivo* model to validate the observed effects. In addition, the potential of increasing EPS production through co-culture strategies is promising, considering the technological and prebiotic benefits associated with bacterial EPS.

## Data availability statement

The datasets presented in this study can be found in online repositories. The names of the repository/repositories and accession number(s) can be found in the article/Supplementary material.

## Author contributions

BDGO and EK: investigation, formal analysis, conceptualization, methodology, writing – original draft, reviewing and editing. RJ-F: funding acquisition, resources, supervision, conceptualization, and formal analysis. VA: supervision, resources, review and editing. All authors contributed to the article and approved the submitted version.

## Funding

This project was funded by the Wilbur A. Gould Food Industries Center and the J.T. “Stubby” Parker Endowment in Dairy Foods at The Ohio State University (Columbus, OH).

## Conflict of interest

The authors declare that the research was conducted in the absence of any commercial or financial relationships that could be construed as a potential conflict of interest.

## Publisher’s note

All claims expressed in this article are solely those of the authors and do not necessarily represent those of their affiliated organizations, or those of the publisher, the editors and the reviewers. Any product that may be evaluated in this article, or claim that may be made by its manufacturer, is not guaranteed or endorsed by the publisher.
