# Supplementary material for: Enhanced probiotic potential of Lactobacillus kefiranofaciens OSU-BDGOA1 through co-culture with Kluyveromyces marxianus bdgo-ym6
Source: Front Microbiol. 2023 Aug 3;14:1236634. doi: 10.3389/fmicb.2023.1236634 (PMC10434783; doi:10.3389/fmicb.2023.1236634)
Supplement: Supplementary file 1 [file Data_Sheet_1.pdf]

Supplementary Material

Enhanced probiotic potential of *Lactobacillus kefiranofaciens* OSU-BDGOA1 through co-culture with *Kluyveromyces marxianus* bdgo-ym6

Brianda D. González-Orozco[1], Erica Kosmerl[1], Rafael Jimenez-Flores[1], Valente B. Alvarez[1]*

*** Correspondence:** Valente B. Alvarez, alvarez.23@osu.com

1 Supplementary Tables

Supplementary Table 1. Information of *Lactobacillus kefiranofaciens* strains used for genome comparison.

Strain	Source	Genome size	CDS number	Accession number
Lactobacillus kefiranofaciens **1207**	Kefir	2.1	2072	NZ_CP061341.1
Lactobacillus kefiranofaciens subsp. *kefiranofaciens* **ZW3**	Kefir grains	2.3	2273	NC_015602.1
Lactobacillus kefiranofaciens subsp. *kefiranofaciens* **LKK75**	Kefir grains	2.3	2251	NZ_CP045033.1
Lactobacillus kefiranofaciens **OSU-BDGOA1**	Kefir grains	2.0	2022	JARJVW01

Supplementary Table 2. Targeted functions of genes mined from *L. kefiranofaciens* OSU-BDGOA1 genome with IslandViewer4.

Function	Gene description	Gene	GenPept Annotation
**Metabolism of carbohydrates**			
**Lactose**	Beta-galactosidase small subunit	lacM	MDF4141715.1
	Lactate dehydrogenase		MDF4143132.1
	Alpha-galactosidase		MDF4141699.1
	UTP--glucose-1-phosphate uridylyltransferase	galU	MDF4142441.1
	UDP-glucose--hexose-1-phosphate uridylyltransferase		MDF4141720.1
	Galactokinase	galK	MDF4141719.1
	Aldose 1-epimerase	galM	MDF4142092.1
	UDP-glucose 4-epimerase GalE	galE	MDF4141714.1
**Glucose**	6-phospho-beta-glucosidase BglA	bglA	MDF4142186.1
	6-phospho-alpha-glucosidase		MDF4142647.1
	PTS glucose transporter subunit IIA		MDF4142456.1
**Others**	Alpha-glucosidase		MDF4142685.1
	Beta-phosphoglucomutase		MDF4142682.1
	PTS sugar transporter subunit IIA		MDF4141547.1
	PTS sugar transporter subunit IIC		MDF4141549.1
	PTS sugar transporter subunit IIB		MDF4141548.1

	PTS system mannose/fructose/sorbose family transporter subunit IID		MDF4141550.1
**Exopolysaccharide production**	Glycerol-3-phosphate cytidylyltransferase	tagD	MDF4142536.1
	Glucose-6-phosphate isomerase	pgi	MDF4142328.1
	PssD/Cps14F family polysaccharide biosynthesis glycosyltransferase	pssD	MDF4143010.1
	Glycosyltransferase		MDF4142924.1
	Glycosyltransferase family 4 protein		MDF4141583.1
	Glycosyltransferase family 2 protein		MDF4142262.1
	Glycosyltransferase family 8 protein		MDF4142922.1
	Exopolysaccharide biosynthesis protein		MDF4142672.1
	EpsG family protein	epsG	MDF4143015.1
	CpsD/CapB family tyrosine-protein kinase		MDF4142673.1
	UDP-N-acetylglucosamine 2-epimerase	wecB	MDF4142446.1
	CpsD/CapB family tyrosine-protein kinase		MDF4142673.1
	Bifunctional UDP-N-acetylglucosamine diphosphorylase/glucosamine-1-phosphate N-acetyltransferase GlmU	glmU	MDF4143090.1
	Oligosaccharide flippase family protein		MDF4141602.1
**Adherence**	Aggregation promoting factor surface protein		MDF4142727.1

	S-layer protein		MDF4143215.1
	S-layer protein		MDF4143216.1
	SLAP domain-containing protein		MDF4142906.1
**Bacteriocin production**	Helveticin J family class III bacteriocin		MDF4142905.1
	Helveticin J family class III bacteriocin		MDF4142904.1
	Class III bacteriocin		MDF4141648.1
	Bacteriocin immunity protein		MDF4142463.1
**Acid resistance**	Glutamate/gamma-aminobutyrate family transporter YjeM	yjeM	MDF4142203.1
	Putative ornithine decarboxylase		MDF4142086.1
	Putative amino acid permeases		MDF4142451.1
	Cation:proton antiporter		MDF4143055.1
	Na+/H+ antiporter NhaC	nhaC	MDF4142515.1
	Tyrosine--tRNA ligase 1	tyrS1	MDF4143064.1
**Bile resistance**	Cyclopropane-fatty-acyl-phospholipid synthase	cfa	MDF4141818.1
	ATP-dependent Clp protease ATP-binding subunit ClpX	clpX	MDF4142206.1
	ATP-dependent Clp protease ATP-binding subunit		MDF4142876.1
	Conjugated bile salt MFS transporter		MDF4142167.1

Supplementary Table 3. Growth fitness of *L. kefiranofaciens* in mono- and co-culture with *K. marxianus* in different media assessed by pH, and CFU/mL at 12 and 48 h.

**Conditions**	Milk aerobiosis (35 °C) 12 and 48 h		MRS anaerobiosis (35 °C) 12 and 48 h		MRS aerobiosis (30 °C) 12 and 48 h		mCGB aerobiosis (30 °C) 12 and 48 h	
**Treatment**	**pH**							
L. kefiranofaciens	4.39	4.0	3.67	3.7	4.13	3.88	4.28	3.82
***L. kefiranofaciens* (Co-culture)**	4.54	3.82	4.03	3.75	4.21	4.03	5.22	5.36
***K. marxianus* (Co-culture)**	5.41	6.34	4.79	4.32	5.86	5.88		
	**log CFU/ml**							
L. kefiranofaciens	8.4	8.4	7.95	8.4	8.8	9.68	7.6	9.2
***L. kefiranofaciens* (Co-culture)**	8.81	8.81	8.43	8.8	8.68	8.3	9.15	8.61
***K. marxianus* (Co-culture)**	5.81	5.81	ND	ND	4.81	5.37	5.37	5.37

2 Supplementary Figures

A

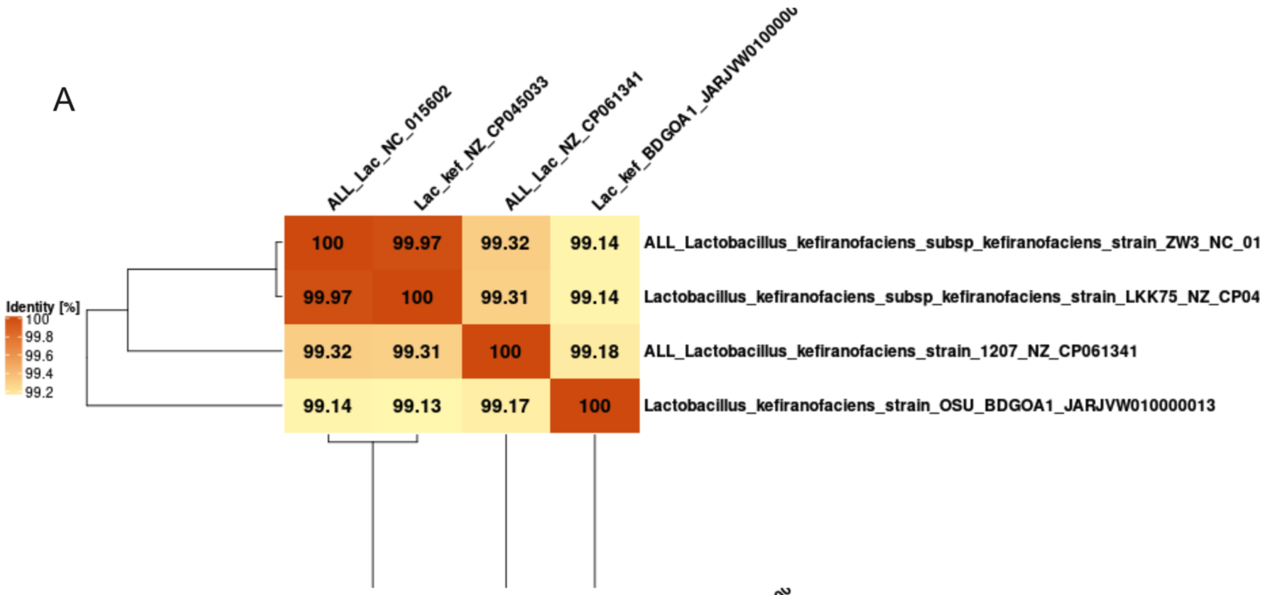
ALL_Lac_NC_015602
Lac_kef_NZ_CP045033
ALL_Lac_NZ_CP061341
Lac_kef_BDGOA1_JARJVW010000
Identity [%]
100
99.8
99.6
99.4
99.2
100 99.97 99.32 99.14
99.97 100 99.31 99.14
99.32 99.31 100 99.18
99.14 99.13 99.17 100
ALL_Lactobacillus_kefiranofaciens_subsp_kefiranofaciens_strain_ZW3_NC_01
Lactobacillus_kefiranofaciens_subsp_kefiranofaciens_strain_LKK75_NZ_CP04
ALL_Lactobacillus_kefiranofaciens_strain_1207_NZ_CP061341
Lactobacillus_kefiranofaciens_strain_OSU_BDGOA1_JARJVW010000013

B

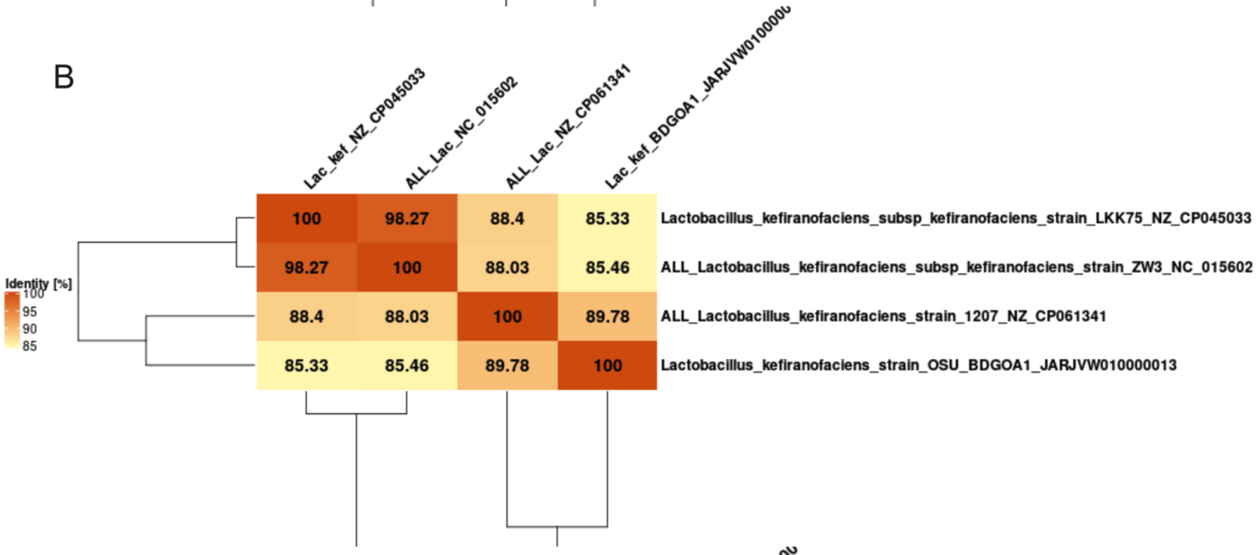
Lac_kef_NZ_CP045033
ALL_Lac_NC_015602
ALL_Lac_NZ_CP061341
Lac_kef_BDGOA1_JARJVW010000
Identity [%]
100
95
90
85
100 98.27 88.4 85.33
98.27 100 88.03 85.46
88.4 88.03 100 89.78
85.33 85.46 89.78 100
Lactobacillus_kefiranofaciens_subsp_kefiranofaciens_strain_LKK75_NZ_CP045033
ALL_Lactobacillus_kefiranofaciens_subsp_kefiranofaciens_strain_ZW3_NC_015602
ALL_Lactobacillus_kefiranofaciens_strain_1207_NZ_CP061341
Lactobacillus_kefiranofaciens_strain_OSU_BDGOA1_JARJVW010000013

C

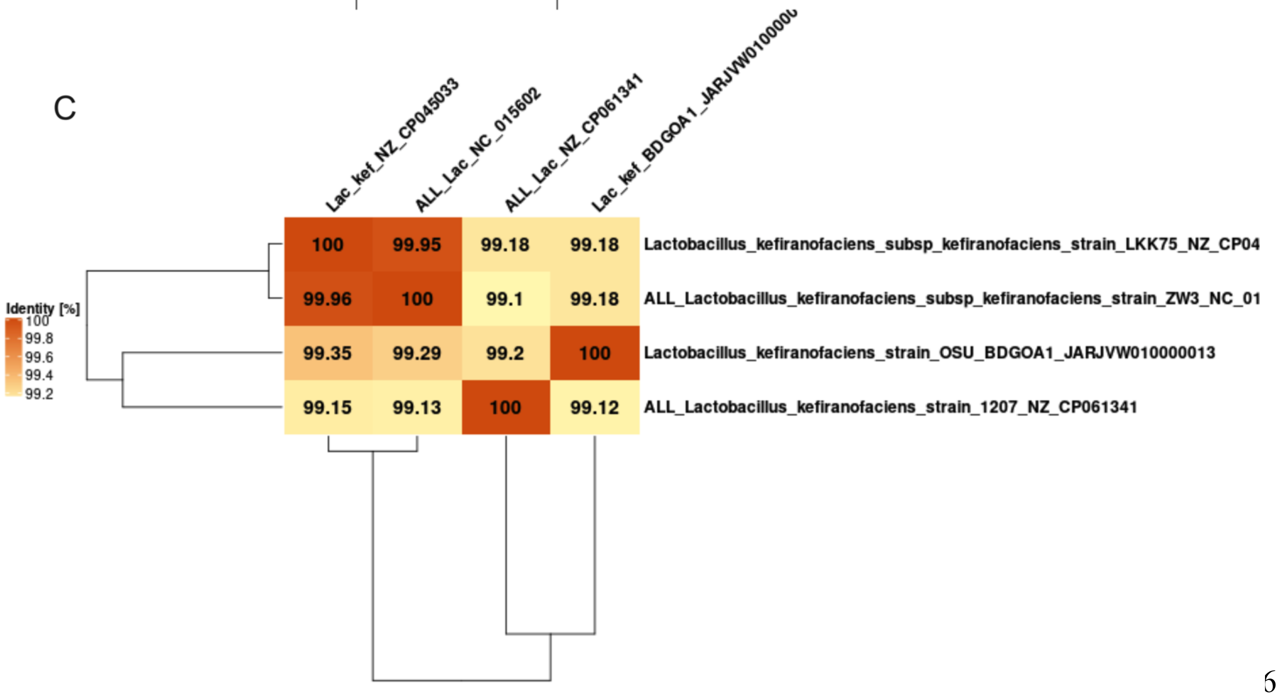
Lac_kef_NZ_CP045033
ALL_Lac_NC_015602
ALL_Lac_NZ_CP061341
Lac_kef_BDGOA1_JARJVW010000
Identity [%]
100
99.8
99.6
99.4
99.2
100 99.95 99.18 99.18
99.96 100 99.1 99.18
99.35 99.29 99.2 100
99.15 99.13 100 99.12
Lactobacillus_kefiranofaciens_subsp_kefiranofaciens_strain_LKK75_NZ_CP04
ALL_Lactobacillus_kefiranofaciens_subsp_kefiranofaciens_strain_ZW3_NC_01
Lactobacillus_kefiranofaciens_strain_OSU_BDGOA1_JARJVW010000013
ALL_Lactobacillus_kefiranofaciens_strain_1207_NZ_CP061341

Supplementary Figure 1. Genomic-based comparison between *Lactobacillus kefiranofaciens* BDGOA1 and closely related strains of the species. *L. kefiranofaciens* OSU-BDGOA1 (reference genome), *L. kefiranofaciens* subsp. *kefiranofaciens* LKK75, *L. kefiranofaciens*1207, and *L. kefiranofaciens* subsp. *kefiranofaciens* ZW3, respectively. Average amino acid identity (AAI) (A), percentage of conserved proteins (POCP) (B), and mean nucleotide identity of orthologous genes (FastANI)(C).

***L. innocua* ATCC 51742**	***E. coli* ATCC 25922**
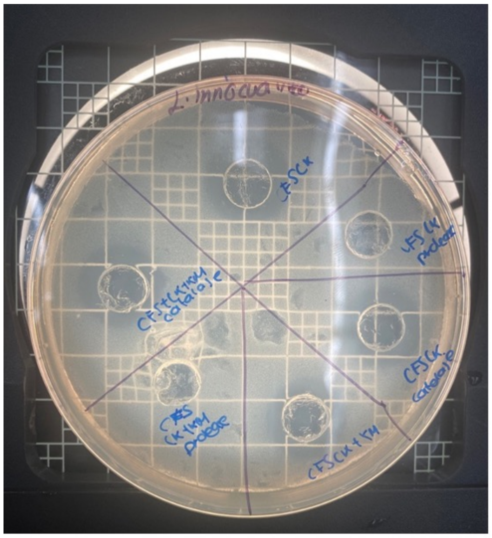	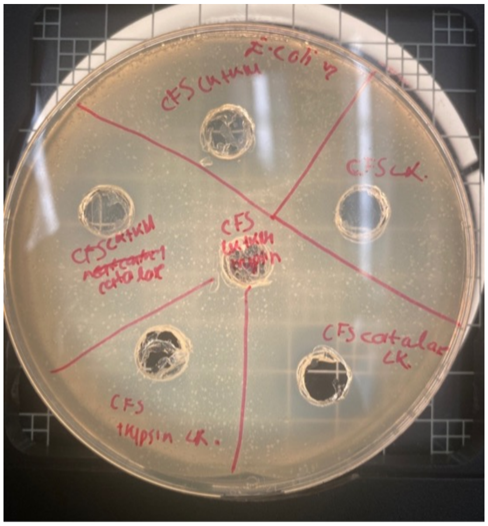

Supplementary Figure 2. Antibacterial activity of cell-free supernatants (CFS) from *L. kefiranofaciens* (LK) in mono- and co-culture with *K. marxianus* (LK-KM) against indicator strains by the agar well diffusion assay**.** Treatments: CFS, CFS + catalase, CFS + trypsin (protease).

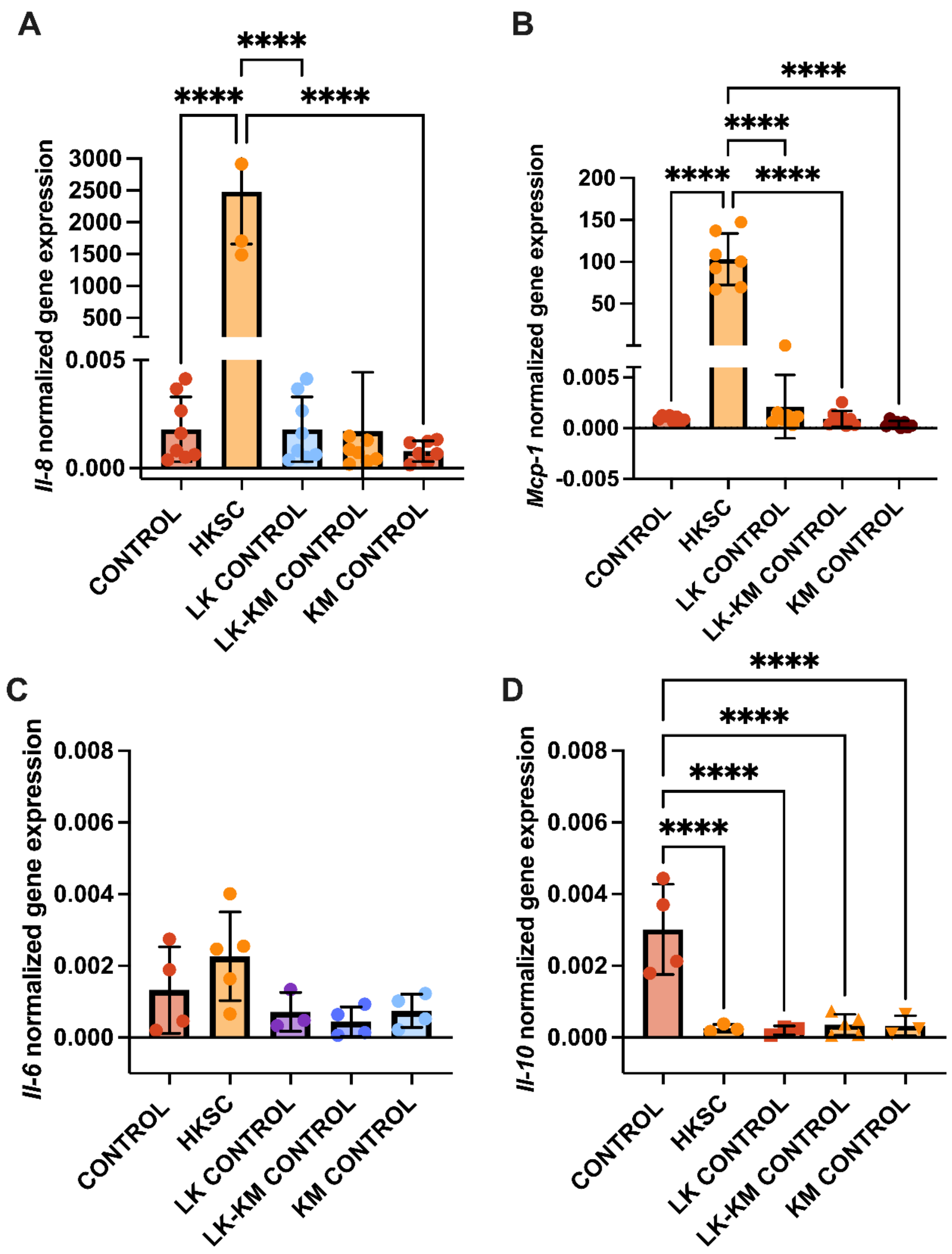
A
****
****
****
Il-8 normalized gene expression
3000
2500
2000
1500
1000
500
0.005
0.000
CONTROL
HKSC
LK CONTROL
LK-KM CONTROL
KM CONTROL
B
****
****
****
****
Mcp-1 normalized gene expression
200
150
100
50
0.005
0.000
-0.005
CONTROL
HKSC
LK CONTROL
LK-KM CONTROL
KM CONTROL
C
Il-6 normalized gene expression
0.008
0.006
0.004
0.002
0.000
CONTROL
HKSC
LK CONTROL
LK-KM CONTROL
KM CONTROL
D
****
****
****
****
Il-10 normalized gene expression
0.008
0.006
0.004
0.002
0.000
CONTROL
HKSC
LK CONTROL
LK-KM CONTROL
KM CONTROL

Supplementary Figure 3. Effect of co-culture (LK-KM) and monocultures (LK, KM) on the expression of cytokines (*ll-8, mcp-1, Il-6, Il-10*) in Caco-2 cells. Values represent the mean + SD of at least three replicates. Statistical difference denoted as $*p < 0.05$, $**p < 0.01$, $****p < 0.0001$ by one-way ANOVA with *post hoc* Tukey test.

frontiers
